# Spatial Transcriptomics of Developing Wheat Seed Reveals Concentric Gene Expression Zones and Subgenome Biased Expression of Key Genes

**DOI:** 10.1111/pbi.70351

**Published:** 2025-09-04

**Authors:** Tori Millsteed, David Kainer, Robert Sullivan, Xiaohuan Sun, Ka Leung Li, Likai Mao, Arlie Macdonald, Robert J. Henry

**Affiliations:** ^1^ Queensland Alliance for Agriculture and Food Innovation (QAAFI) University of Queensland St Lucia Queensland Australia; ^2^ ARC Centre of Excellence for Plant Success in Nature and Agriculture University of Queensland St Lucia Queensland Australia; ^3^ Queensland Brain Institute University of Queensland St Lucia Queensland Australia; ^4^ MGI Australia Herston Queensland Australia

**Keywords:** embryo, homeologs, marker genes, pericarp, seed, STOmics

## Abstract

Gene expression of developing seeds drives essential processes such as nutrient storage, stress tolerance and germination. However, the spatial organisation of gene expression within the complex structure of the seed remains largely unexplored. Here we report the use of the STOmics spatial transcriptomics platform to visualise spatial expression patterns in the wheat ( *Triticum aestivum* ) seed at the critical period of grain filling in mid‐seed development. We analysed > 4 000 000 spatially resolved transcripts, achieving subcellular resolution of transcript localisation across multiple tissue domains, and identified gene expression clusters linked to eight functional cellular groups. Notably, our analysis characterised four distinct clusters within the endosperm, appearing in concentric zones from the inner to outer regions of the grain, and identified novel marker gene candidates for the clusters found. We further investigated known tissue‐specific genes and identified subgenome biased expression for the genes puroindoline‐B, metallothionein protein, and α‐amylase/subtilisin inhibitor. These findings provide new detail about gene expression across and within different functional cellular groups of the developing seed and demonstrate that spatial transcriptomics could further our understanding of subgenome differences in polyploid plants. Furthermore, this dataset offers a significant resource of spatial gene expression in the 14 DPA wheat seed and will support future research on grain filling.

## 1 Introduction

The development of seeds is driven by spatiotemporally distinct gene expression, which impacts seed size and vigour of the resulting plant (Chaudhury et al. 2001; Ohto et al. 2007; Li et al. 2014; Bizouerne et al. 2021; Pelletier et al. 2024). Key traits such as nutrient storage, stress tolerance, germination, and the nutritional properties of seeds are influenced by gene expression in specific tissues and cell regions, and the interactions between these spatial groups (Dwivedi et al. 2021). Therefore, understanding gene expression in the complex structure of the seed is significant to plant breeders, geneticists, and ecologists in the context of optimisation for food security and climate change (Dwivedi et al. 2021; Theissinger et al. 2023). While transcriptomics analyses have helped identify key genes involved in these processes, they have lacked the spatial detail needed to complete the picture of tissue‐specific gene expression driving development. The emergence of spatial transcriptomics technologies has allowed for gene expression data to be placed into detailed spatial resolution, providing new insights about the genes underpinning agronomically and ecologically important traits (Yin et al. 2023).

Seed development is a highly coordinated process that takes place in different stages across various tissues (Bechtel et al. 2009). Development begins with double fertilisation resulting in the formation of the endosperm and early differentiation of the embryo. For monocots, the endosperm is the predominant structure of the seed and the source of energy reserves used by the embryo at germination (Brown and Lemmon 2007). For cereal crops such as wheat ( *Triticum aestivum* ), endosperm development is also known as the grain filling stage and is the main determinant of yield (Sabelli and Larkins 2009). Due to its agronomic importance, grain filling has been extensively studied and it is estimated to peak between 14 and 20 days post anthesis (DPA). Endosperm development involves sequential stages of cell division and differentiation, and accumulation of storage reserves such as starch and proteins (Shewry et al. 2012). The process of accumulation occurs in layers with a higher protein content directed to the sub‐aleurone and increasing starch deposition in the central endosperm (Shewry et al. 2020). Additionally, sugars and amino acids supplied via the crease are transported outwards towards the sub‐aleurone layer and exhibit a decreasing gradient away from the crease (Ugalde and Jenner 1990). Throughout development, wheat seeds are also photosynthetically active via the chlorophyll‐rich tube‐ and cross‐cell layers of the pericarp. This observation has led to the proposal that seeds utilise already respired carbon accumulating in the endosperm for photosynthesis, through a multicellular mechanism linking the endosperm and the tube‐ and cross‐cells (Rangan et al. 2024). This biochemistry would be significant to yield and stress tolerance; however, it is not yet characterised at the gene network level. Despite the established knowledge of the physiological development patterns of the wheat seed, there is a need for more detailed exploration of the spatiotemporal gene expression underlying these processes.

The polyploid genome of wheat, a hexaploid plant, adds further complexity to the study of gene expression. Modern hexaploid bread wheat resulted from hybridisation between tetraploid wheat, *Triticum turgidum* (AABB subgenomes), and diploid *Aegilops tauschii* (DD subgenome) approximately 10 000 years ago (Peng et al. 2011). Polyploid plants tend to have distinct traits such as larger seeds or improved vigour compared to their ancestral lines (Chan et al. 2022). Additionally, species with higher ploidy levels are reported to exhibit enhanced tolerance to biotic and abiotic stressors (Li et al. 2024). Polyploidization events often result in asymmetrical expression between subgenomes and unbalanced contribution to particular biological processes (Feldman et al. 2012). For example, in wheat, the D subgenome is reported to be more highly expressed in response to stressors (Zheng et al. 2022; Powell et al. 2016), which has consequences for seed development. Therefore, understanding subgenome expression in wheat is agronomically important, yet very little research has considered spatial subgenome expression biases. As different tissues and cellular groups influence different developmental processes, investigating their spatial context would offer new insight about how subgenomes contribute to seed development in polyploid plants.

In the post‐genomics era of genetics research, transcriptomics provides a more detailed and more cost‐effective method to study gene activity in plants, uncovering countless genes influencing important traits. However, the common techniques of bulk RNA‐seq and single cell RNA sequencing (scRNA‐seq) lack the ability to analyse gene expression of single cells in their entire spatial context. The recent evolution of spatial transcriptomics addresses this gap (Giacomello 2021). A number of spatial transcriptomics platforms have emerged with differing techniques to visualise gene expression, and at different resolutions. The technique of in situ capture and scRNA‐seq are common to several platforms, allowing the entire mRNA contents of a tissue section to be sequenced with spatial information, and mapped back into the original tissue image (Yin et al. 2023). In recent years, this technique has been used in the study of *Arabidopsis thaliana* leaves (Xia et al. 2022), the barley grain (Peirats‐Llobet et al. 2023), the maize kernel (Fu et al. 2023) and ear (Wang et al. 2024), soybean nodules (Liu et al. 2023), tomato calli (Song et al. 2023), wheat inflorescences (Long et al. unpublished), and recently in the developing wheat grain (Li et al. 2025). Li et al. (2025) analysed the spatial transcriptome of the developing wheat grain at 4, 8, and 12 days post anthesis (DPA), and identified 10 distinct cell types and associated marker genes. The seed sections were cut longitudinally through the ventral and dorsal sides, providing a viewpoint of spatial expression parallel to the midline of the seed structure. Their study provided the first comprehensive spatiotemporal dataset of early wheat seed development and forms the foundation for further work to be undertaken at different time points and with new genetic targets.

Here we utilised STOmics scStereo‐seq to analyse the spatial transcriptome of the 14 DPA wheat seed. Our study provides the continuation of the developmental time points highlighted by Li et al. (2025), focusing on the peak of grain filling in mid‐seed development. We also investigated the alternative view of spatial gene expression with tissue sections cut longitudinally through the lateral sides of the seed, resulting in cross‐sections parallel with the dorsal and ventral sides of the seed structure. Our study aimed to assess spatial gene expression patterns in the complex structure of the seed during grain filling, as well as subgenome‐biased expression of key genes.

## 2 Results

STOmics scStereo‐seq was used to measure spatial gene expression in the developing wheat seed. Sections of 14 DPA seeds were cut in a cryostat to a thickness of 20 μm and mounted onto the STOmics transcriptomics assessment chips, covered in an array of spatially tagged DNA nanoballs (DNBs). The contents of the chips were sequenced, aligned to the reference genome IWGSC CS RefSeq v2.1 (Zhu et al. 2021), and mapped back into their original locations. The resulting spatially tagged gene expression matrix was also overlaid with fluorescent microscope images of the original tissue to place the spatial expression patterns within the cell and tissue architecture. Due to challenges adhering the tissue sections to the chip surface, the most intact section was chosen as the focus for this study, though some folding of the tissue is present. Data from replicate chips have been used to support our findings where possible.

### 2.1 Gene Expression Matrix Reveals Areas of High Gene Activity That Align With Key Tissues

**FIGURE 1:**
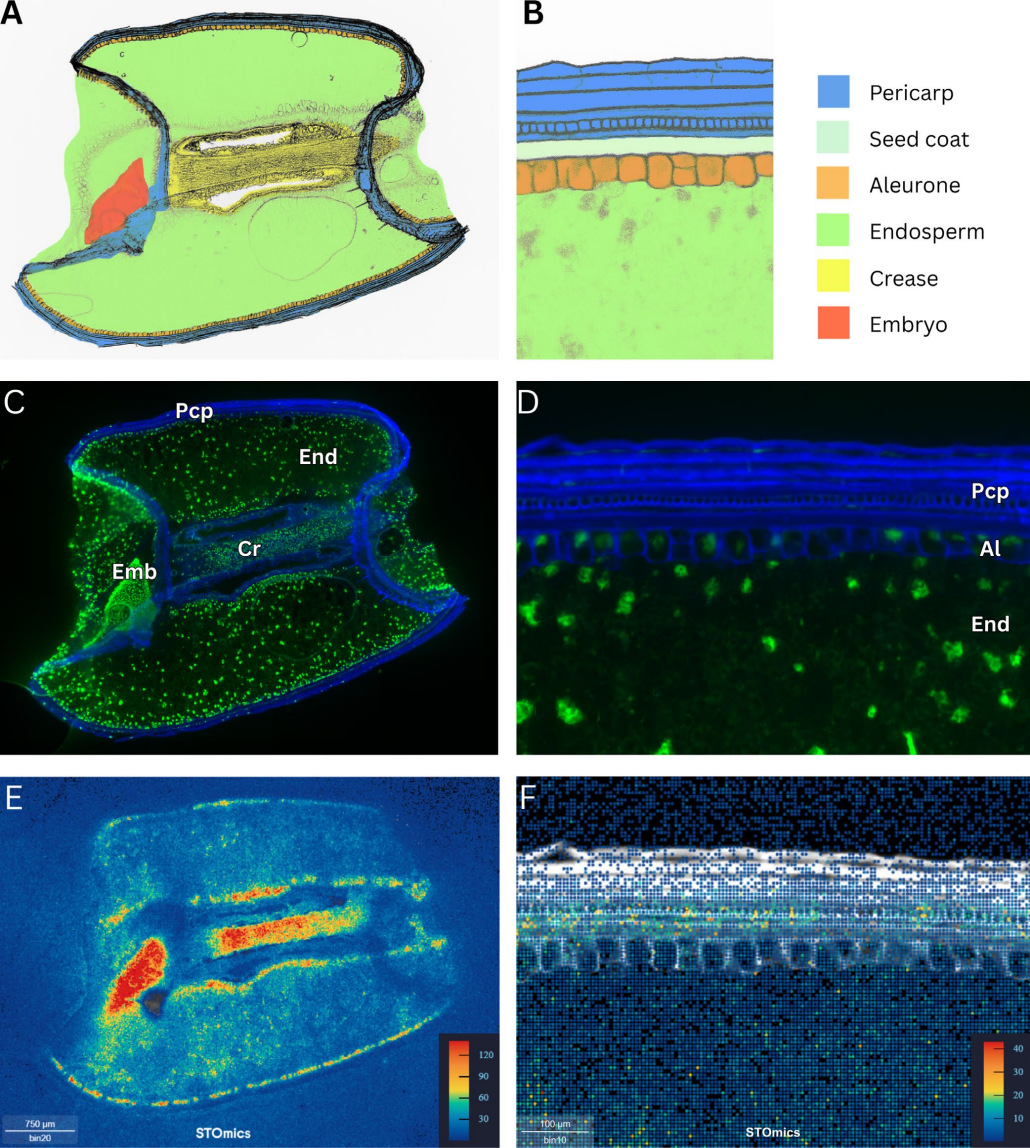
Diagrammatical images, fluorescent microscope images and gene expression heat maps of the whole wheat seed section, and enhanced detail of the outer cell layers, at 14 days post anthesis (DPA). (A) Diagrammatical representation of wheat seed section showing the pericarp, aleurone, endosperm, crease and embryo tissues represented by different colours, indicated in key. (B) Diagrammatical representation of enhanced detail of pericarp, seed coat, aleurone and endosperm tissues, represented by different colours, indicated in key. (C) Fluorescent microscope image of wheat seed section, with cell walls in blue and cell nuclei in green, showing pericarp (Pcp), endosperm (End), embryo (Emb) and crease (Cr) tissues. (D) Fluorescent microscope image showing enhanced detail of pericarp, aleurone (Al) and endosperm tissues. (E) Gene expression heat map of wheat seed section showing areas of highest gene expression in red and lowest gene expression in blue (in counts of transcripts). (F) Gene expression heat map with enhanced detail of pericarp, aleurone and endosperm tissues, showing the specific spatial distribution of gene expression in these cell layers. Scale bars in C, E = 750 μm and D, F = 100 μm.

Fluorescent microscope images of the seed sections highlighted the tissue structure. Well‐defined cell walls of the pericarp and aleurone layers were visible in the blue channel, and a scattered array of cell nuclei of varying sizes in the endosperm, aleurone, and pericarp, stained with the Qubit ssDNA reagent, were visible in the green channel (Figure 1C,D). The gene expression matrix was visualised as a heat map which depicted areas of low expression in dark blue and areas of high expression in red. The areas of highest gene expression were in the embryo, the crease, the inner endosperm, and the pericarp (Figure 1E,F). Additionally, gene expression levels were not symmetrical on both sides of the seed, with both the pericarp and endosperm on one side (lower in our figure) exhibiting greater expression levels than the other side.

For the STOmics chip containing the most intact tissue section, there were 92 046 597 DNBs located beneath the tissue area. Of these, 3 273 120 DNBs successfully captured 4 244 343 MIDs (unique molecular identifiers) at subcellular resolution, resulting in an average of 1.3 MIDs from an average of 1.09 genes per DNB (Figure S2). The sparseness of the capture array was alleviated by grouping adjacent DNBs into 20 × 20 bins (Bin20) where each Bin20 contained an average of 18.47 MIDs from 14 genes. For the replicates analysed, there were between 3 237 120 and 11 599 308 DNBs that successfully captured mRNA beneath the tissue areas, resulting in an average capture array of 1.30–1.57 MIDs per DNB (Figures S2, S4, S6).

### 2.2 Gene Expression Clusters Appear in Concentric Zones That Match Tissue Areas

**FIGURE 2:**
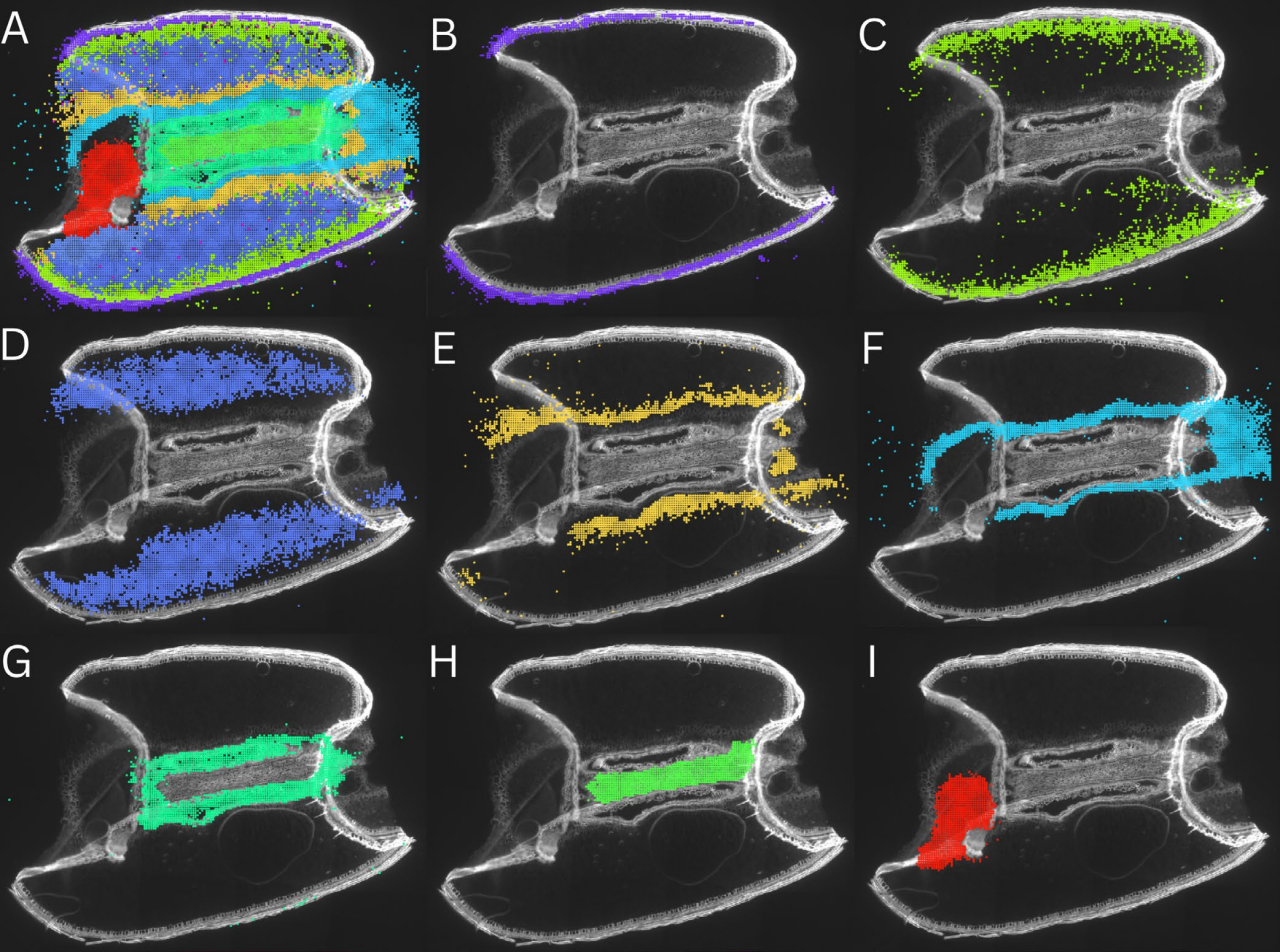
Gene expression clusters identified by Spatial Leiden network analysis in 14 days post anthesis (DPA) wheat seed section. (A) Visualisation of all gene expression clusters identified, (B) cluster aligned with pericarp tissue, (C) cluster aligned with sub‐aleurone layer of endosperm, (D) cluster aligned with central endosperm, (E) cluster aligned with inner endosperm, (F) cluster aligned with transfer cells, (G) cluster aligned with outer crease cells, (H) cluster aligned with inner crease cells and (I) cluster aligned with embryo tissue.

Spatial Leiden clustering was performed on the expression data from each chip at a resolution of Bin50. A total of 8 clusters were identified and linked to functional cellular groups (Figure 2). These were the pericarp (Figure 2B), the sub‐aleurone (Figure 2C), the central endosperm (Figure 2D), the inner endosperm (Figure 2E), the transfer cells (Figure 2F), the outer crease (Figure 2G), the inner crease (Figure 2H) and the embryo (Figure 2I). While clusters B, G, H, and I were linked to a single tissue type or cellular group, clusters C, D, E, and F were all located within the same tissue area, the endosperm. The sub‐aleurone cluster formed the outermost layer of the endosperm and followed the ring‐like shape of the outer boundary of the tissue section, with some expression observed within the central endosperm region. The central endosperm cluster covered the largest area of the seed section and, while strongly localised to the central endosperm, also exhibited some expression within the areas of the neighbouring clusters. The inner endosperm and transfer cell clusters also appeared in distinct, concentric layers, encircling the crease at the centre of the tissue section. The transfer cell cluster was one of the areas of greatest gene expression in the seed, and the presence of the inner endosperm cluster, distinct from the central endosperm and transfer cells, highlighted an area of unique gene expression in the endosperm that has not previously been characterised. Similar gene expression clusters appearing in concentric zones, and with multiple distinct layers in the endosperm, were also identified in the replicate chips (Figure S8).

### 2.3 Expression of Known Tissue‐Specific Genes Confirms Data Accuracy

**FIGURE 3:**
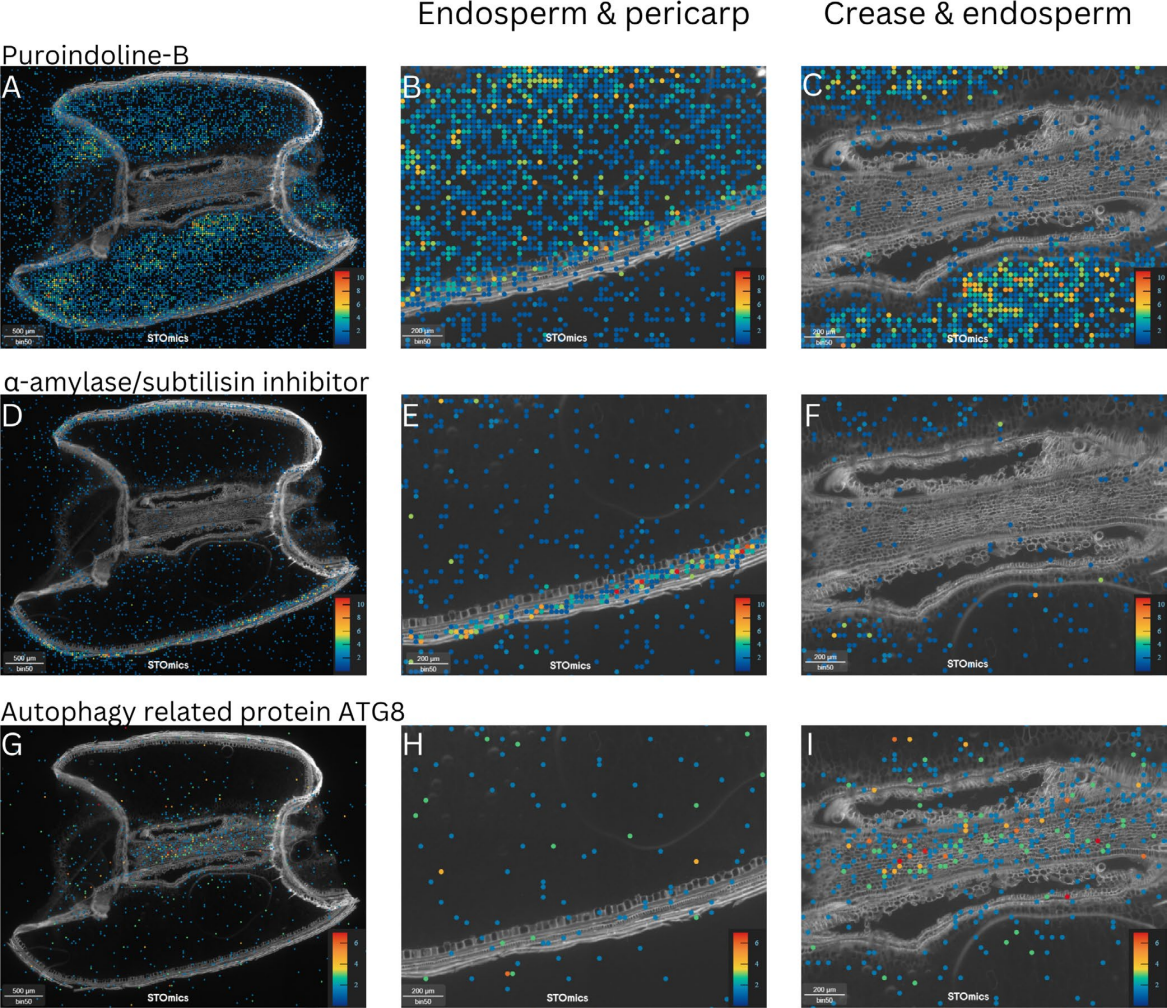
Spatial expression patterns of known tissue‐specific genes across various tissues in a 14 days post anthesis (DPA) wheat seed section. (A) Puroindoline‐B expression in entire seed section, (B) Puroindoline‐B expression in endosperm and pericarp, (C) Puroindoline‐B expression increase and endosperm, (D) α‐amylase/subtilisin inhibitor expression in entire seed section, (E) α‐amylase/subtilisin inhibitor expression in endosperm and pericarp, (F) α‐amylase/subtilisin inhibitor expression increase and endosperm, (G) Autophagy related protein ATG8 expression in entire seed section, (H) Autophagy related protein ATG8 expression in endosperm and pericarp, (I) Autophagy related protein ATG8 expression increase and endosperm. Scale bars in A, D and G = 500 μm/50 bins and in B, C, E, F, H and I = 200 μm. The heat map scales located in each image shows the highest expression in red and the lowest expression in blue.

We analysed the spatial expression of known tissue‐specific genes to validate the robustness of our data and confirm their expression in the expected tissue types. This included genes known, or expected, to be expressed in the pericarp, endosperm, and embryo tissues. The genes for puroindoline‐B were investigated, as they are related to wheat endosperm hardness and known to be highly expressed in the endosperm and aleurone cells (Nirmal et al. 2016). This was confirmed by our gene expression matrix, which showed high levels of expression in the endosperm compared to other tissue types, particularly when compared to the crease (adjusted approximate permutation tests *p* < 0.01). However, our data also showed that these genes were expressed at some level in all tissue types (Figure 3A–C). *TaNAC019*, a wheat transcription factor involved in starch biosynthesis in the endosperm (Gao et al. 2021) was also investigated, and expression levels were found to be generally low but appeared to be specific to the endosperm (Figure S9). Expression levels of the *TaNAC019* genes varied compared to those reported by Gao et al. (2021); however, they were consistent with the findings that their expression is low until around 18–20 DPA. Similarly, wheat transcription factor *TabZIP28*, also known to be involved in starch biosynthesis (Song et al. 2020), appeared to be mostly expressed in the endosperm (Figure S10), but expression levels were too low for this to be considered significant.

Metallothionein protein genes were assessed as these were expected to be embryo specific (Kawashima et al. 1992). These were most highly expressed in the embryo, followed by the pericarp and crease, and then lower expression in the endosperm tissue (all adjusted approximate permutation tests *p* < 0.01) (Figure S11). The genes encoding the EM promoter protein were also investigated as they are known to be specifically expressed in the embryo and the aleurone layer (Furtado and Henry 2005). These genes were most highly expressed in the embryo and the pericarp, and they also exhibited some lower expression in the endosperm and crease (all adjusted approximate permutation tests *p* < 0.01) (Figure S12).

Genes encoding α‐amylase/subtilisin inhibitor were investigated, as they are highly expressed in the barley pericarp (Furtado et al. 2003). These genes exhibited a pattern of strongest expression in the pericarp in wheat (all adjusted approximate permutation tests *p* < 0.01), as well as expression at lower levels throughout the endosperm, crease, and embryo (Figure 3D–F). The genes for pyruvate orthophosphate dikinase were also assessed as pericarp specific, due to their role in photosynthesis (Rangan et al. 2016). These genes were most highly expressed in the pericarp and the embryo (all adjusted approximate permutation tests *p* < 0.01), but also showed some dispersal in the endosperm (Figure S13). Lastly, the genes encoding autophagy‐related protein ATG8 were investigated. These genes are highlighted in the literature as being involved in programmed degradation of pericarp cells (Li et al. 2021). While there was some expression of these genes in the pericarp layer, expression appeared to be more strongly localised to the crease region of the grain, with some expression in the endosperm at similar levels to the expression seen in the pericarp (Figure 3G–I) (Table 1). However, these observed differences were not found to be statistically significant.

**TABLE 1:** Reported and observed spatial expression patterns of known tissue‐specific genes in a 14 days post anthesis (DPA) wheat seed section. Table includes gene name and/or function, IWGSC Chinese Spring Refseq v2.1 gene ID, subgenome of homeologs, the reported spatial expression pattern from the literature, the observed spatial expression pattern in the data presented, and the overall homeolog expression (in MID counts) in the replicate seed sections analysed.

Gene name/function	Gene ID	Sub‐genome	Reported spatial expression	Observed spatial expression in our data	Overall homeolog expression (MID count)		
					Rep 1	Rep 2	Rep 3
Puroindoline‐B	*TraesCS5A03G0006700*	A	Endosperm/aleurone	Endosperm/aleurone	2836	15 021	3817
	*TraesCS5B03G0006500*	B			7387	35 537	10 749
	*TraesCS5D03G0008100*	D			24 634	97 257	34 577
*TaNAC019*	*TraesCS3A03G0172000*	A	Endosperm	Endosperm	238	373	226
	*TraesCS3B03G0216600*	B			0	0	0
	*TraesCS3D03G0154500*	D			82	262	86
*TabZIP28*	*TraesCS2A03G0291300*	A	Endosperm	Endosperm/crease	153	219	146
	*TraesCS2B03G0409300*	B			287	383	200
	*TraesCS2D03G0306500*	D			205	242	148
Metallothionein‐protein		A	Embryo	Embryo/pericarp/crease	0	0	0
	*TraesCS1B03G0086800*	B			2131	585	785
	*TraesCS1D03G0066100*	D			21 397	8362	9896
EM promoter protein	*TraesCS1A03G0887200*	A	Embryo	Embryo/pericarp/crease	1543	1617	893
	*TraesCS1B03G1037600*	B			2100	1292	819
	*TraesCS1D03G0862200*	D			1439	894	550
α‐amylase/subtilisin inhibitor	*TraesCS2A03G0911300*	A	Pericarp	Endosperm	123	458	109
	*TraesCS2B03G1001100*	B		Pericarp/endosperm	4286	13 229	3443
	*TraesCS2D03G0850900*	D			2730	13 649	4056
Pyruvate orthophosphate dikinase	*TraesCS1A03G0652400*	A	Pericarp	Pericarp/embryo/endosperm	1380	3111	1038
	*TraesCS1B03G0741000*	B			609	1658	579
	*TraesCS1D03G0610200*	D			1418	2625	884
Autophagy related protein ATG8	*TraesCS2A03G0482400*	A	Pericarp	Crease/endosperm/pericarp	533	958	371
	*TraesCS2B03G0601600*	B			711	1021	432
	*TraesCS2D03G0485800*	D			690	1123	515

### 2.4 Subgenome Biased Expression Identified for Some Key Genes

**FIGURE 4:**
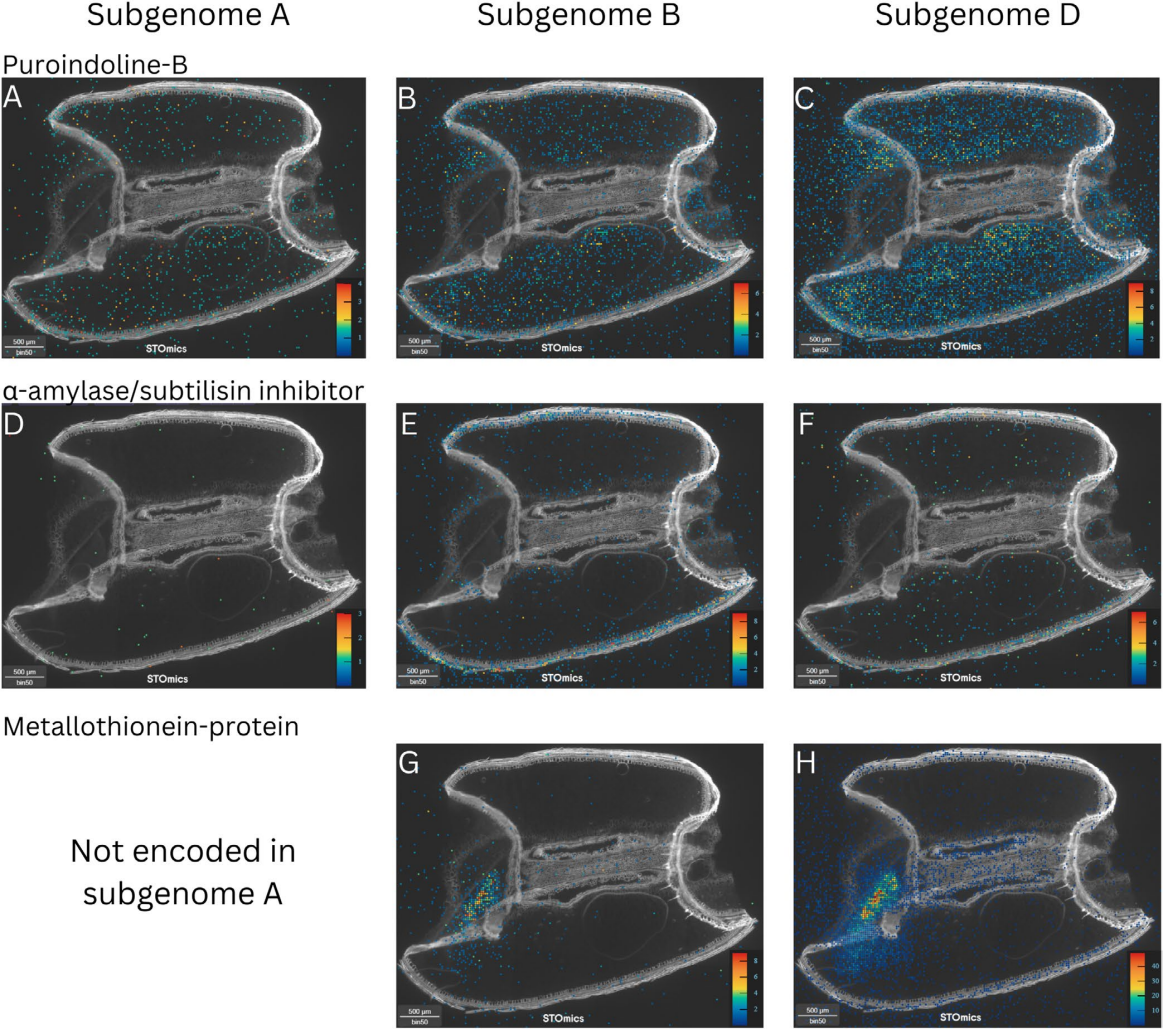
Examples of subgenome biased expression patterns between homeologs of known tissue‐specific genes in 14 days post anthesis (DPA) wheat seed section. (A) Puroindoline‐B subgenome A homeolog expression, (B) Puroindoline‐B subgenome B homeolog expression, (C) Puroindoline‐B subgenome D homeolog expression, (D) α‐amylase/subtilisin inhibitor subgenome A homeolog expression, (E) α‐amylase/subtilisin inhibitor subgenome B homeolog expression, (F) α‐amylase/subtilisin inhibitor subgenome D homeolog expression, (G) Metallothionein‐protein subgenome B homeolog expression, (H) Metallothionein‐protein subgenome D homeolog expression. Scale Bars = 500 μm/50 bins. The heat map scales located in each image shows the highest expression in red and the lowest expression in blue.

Subgenome biased expression was identified for some of the genes analysed. For puroindoline‐B, the subgenome D homeolog was significantly more highly expressed overall than the subgenome A and B homeologs (Figure 4A–C), with 24 634 MIDs compared to 7387 and 2836 respectively (all adjusted approximate permutation tests *p* < 0.01). The A and B homeologs were also found to be significantly differently expressed (all adjusted approximate permutation tests *p* < 0.01). Similarly, for metallothionein protein, the subgenome D homeolog was expressed at significantly higher levels than the subgenome B homeolog overall (Figure 4G,H), with 21 397 MIDs compared to 2131 MIDs (all adjusted approximate permutation tests *p* < 0.01). There was also notably no subgenome A homeolog of this metallothionein protein isoform present in the reference genome (Zhu et al. 2021).

The genes for α‐amylase/subtilisin inhibitor exhibited subgenome biased spatial expression (Figure 4D–F). The subgenome A homeolog was expressed at significantly lower levels overall than the subgenome B and D copies, with 123 MIDs compared to 4286 and 2730 MIDs respectively (all adjusted approximate permutation tests *p* < 0.01). The subgenome A homeolog also appeared to be nearly only expressed in the endosperm, though this observed difference was not found to be statistically significant. Conversely, the subgenome B and D homeologs were significantly more highly expressed in the pericarp tissue than any other tissue type (all adjusted approximate permutation tests *p* < 0.01).

### 2.5 Novel Marker Genes Identified for Gene Expression Clusters

**FIGURE 5:**
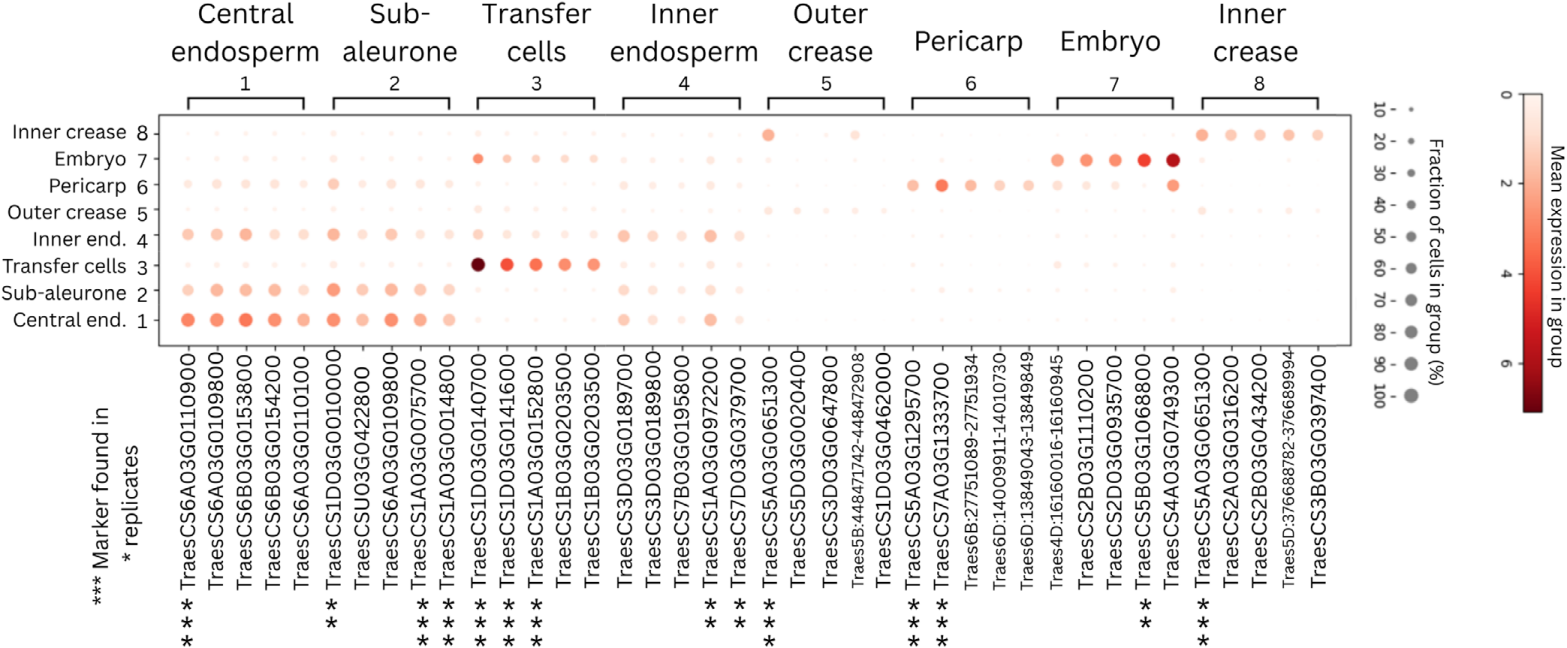
Marker genes plot of the five strongest marker gene candidates for each gene expression cluster, and alignments depicting the expression of each marker gene in other clusters. Where dot size represents the percentage of cells in the respective cluster that a gene is found in, and dot colour represents the mean expression (in MID counts) of the gene in those cells, corresponding to the scale on the left. Asterisk (*) represent the number of times a gene was identified as a marker candidate for its respective cluster in the three replicate seed sections analysed.

The five strongest marker gene candidates for each cluster were identified (Figure 5). Strong marker genes were identified for the pericarp, the crease, the embryo and the transfer cells, while a number of marker genes were identified for the endosperm but were not specific to a particular endosperm cluster. For the pericarp, the strongest marker gene candidates were TraesCS7A03G1333700 (26 kDa endochitinase 2) and TraesCS5A03G1295700 (non‐specific lipid‐transfer protein 2P‐like), which were nearly exclusively expressed in the pericarp (Figure 5), and were also identified as marker genes for this tissue in the replicate chips (Figures S20 and S21). The strongest marker gene candidate for both the inner and outer crease clusters was TraesCS5A03G0651300 (induced stolen tip protein (TUB8)) which was also supported by the replicates (Figures S20 and S21). For the embryo cluster, the strongest marker gene was TraesCS5B03G1068800 (cupincin‐like) and was also identified as a marker for this tissue in the replicate chips (Figure S21).

The strongest marker genes for the transfer cells cluster were TraesCS1D03G0140700, TraesCS1D03G0141600, and TraesCS1A03G0152800, which were identified in all replicates (Figure 5; Figures S20 and S21). The gene TraesCS1D03G0140700 was the most highly expressed in the gene expression matrix overall, and the three were highly similar (94.75%–97.95% nucleotide similarity) (Sayers et al. 2024); however, all three are ‘uncharacterised’ in the reference genome (Zhu et al. 2021). These genes were predicted to encode Bifunctional inhibitor/plant lipid transfer proteins/seed storage helical domain‐containing proteins by automatic annotation (Bateman et al. 2024). The most similar gene to TraesCS1D03G0140700 that is characterised in the reference genome, with approximately 94% similarity, is known as Endosperm transfer cell specific PR60 precursor (Zhu et al. 2021). Additionally, Blastn analysis found a similar gene in the Emmer wheat genome known as putative lipid transfer protein precursor (PR60) gene (accession number FJ459807.1), and BLASTx analysis revealed a structurally similar protein in the *Arabidopsis thaliana* genome known as Bifunctional inhibitor/lipid‐transfer protein/seed storage 2S albumin superfamily protein (accession number 834904), with approximately 90% similarity (Sayers et al. 2024). Therefore, we have inferred that these marker genes are most likely lipid transfer protein genes.

All marker gene candidates identified for the central endosperm, sub‐aleurone and inner endosperm clusters were specific to the endosperm but were expressed at similar levels across neighbouring endosperm clusters (Figure 5). These included genes for α‐, β‐ and γ‐gliadins and α‐amylase/trypsin inhibitors (Table 2).

**TABLE 2:** Marker gene candidates identified for each gene expression cluster, including the cluster name, the IWGSC Chinese Spring Refseq v2.1 gene ID, the gene name/function, and the number of times a gene was identified as a marker candidate for the respective cluster across the three replicate seed sections analysed (*).

Cluster	Gene ID	Gene name/function	Marker gene for this tissue area in * replicates
(1) Central endosperm	*TraesCS6A03G0110900*	Alpha/beta‐gliadin	***
	*TraesCS6A03G0109800*	Alpha/beta‐gliadin	*
	*TraesCS6B03G0153800*	Alpha/beta‐gliadin	*
	*TraesCS6B03G0154200*	Alpha/beta‐gliadin	*
	*TraesCS6A03G0110100*	Alpha/beta‐gliadin	*
(2) Sub‐aleurone	*TraesCS1D03G0010000*	Gamma‐gliadin	**
	*TraesCSU03G0422800*	Alpha/beta‐gliadin	*
	*TraesCS6A03G0109800*	Alpha/beta‐gliadin	*
	*TraesCS1A03G0075700*	Gamma‐gliadin	***
	*TraesCS1A03G0014800*	Vacuolar protein sorting‐associated protein 60.1	***
(3) Transfer cells	*TraesCS1D03G0140700*	Uncharacterised	***
	*TraesCS1D03G0141600*	Uncharacterised	***
	*TraesCS1A03G0152800*	Uncharacterised	***
	*TraesCS1B03G0203500*	Uncharacterised	*
	*TraesCS1B03G0203500*	Uncharacterised	*
(4) Inner endosperm	*TraesCS3D03G0189700*	Alpha‐amylase inhibitor 0.19	*
	*TraesCS3D03G0189800*	Alpha‐amylase inhibitor 0.19‐like	*
	*TraesCS7B03G0195800*	Alpha‐amylase/trypsin inhibitor CM2‐like	*
	*TraesCS1A03G0972200*	Purothionin A	**
	*TraesCS7D03G0379700*	Alpha‐amylase/trypsin inhibitor CM1‐like	**
(5) Outer crease	*TraesCS5A03G0651300*	Induced stolen tip protein TUB8	***
	*TraesCS5D03G0020400*	Photosystem II protein D1‐like	*
	*TraesCS3D03G0647800*	BURP domain‐containing protein 3	*
	*Traes5B:448471742–448 472 908*	Skin secretory protein xP2	*
	*TraesCS1D03G0462000*	photosystem II protein D1‐like	*
(6) Pericarp	*TraesCS5A03G1295700*	NON‐specific lipid‐transfer protein 2P‐like	***
	*TraesCS7A03G1333700*	26 kDa endochitinase 2	***
	*Traes6B:27751089–27 751 934*	Glycine rich cell wall structural protein	*
	*Traes6D:14009911–14 010 730*	Glycine rich cell wall structural protein	*
	*Traes6D:13849043–13 849 849*	Glycine rich cell wall structural protein	*
(7) Embryo	*Traes4D:16160016–16 160 945*	Oleosin 18 kDa‐like	*
	*TraesCS2B03G1110200*	Oleosin 16 kDa	*
	*TraesCS2D03G0935700*	Oleosin 16 kDa‐like	*
	*TraesCS5B03G1068800*	Cupincin	**
	*TraesCS4A03G0749300*	63 kDa globulin‐like protein	*
(8) Inner crease	*TraesCS5A03G0651300*	Induced stolen tip protein TUB8	***
	*TraesCS2A03G0316200*	Uncharacterised	*
	*TraesCS2B03G0434200*	Suppressor of disruption of TFIIS‐like	*
	*Traes5D:376688782–376 689 994*	Predicted GPI‐anchored protein 58	*
	*TraesCS3B03G0397400*	Gibberellin 2‐beta‐dioxygenase 3	*

## 3 Discussion

Transcriptomics analyses of the wheat seed have previously uncovered key genes involved in seed development, stress tolerance, pathogen resistance, baking quality, and overall yield (Yu et al. 2016; Powell et al. 2016; Rangan et al. 2017; Henry et al. 2018; Rangan et al. 2020; Ahmadi‐Ochtapeh et al. 2024). These studies have advanced optimisation of the wheat crop as a food resource; however, they have lacked the capacity to link genes to functional cellular groups in their detailed spatial context. Li et al. (2025) published the first comprehensive spatiotemporal dataset of early seed development in wheat and identified 10 distinct cell types and associated marker genes. The present study provides a biologically significant resource of spatial gene expression at mid‐seed development, which will support future research on the processes contributing to grain filling. Here, spatial transcriptomics revealed gene expression clusters linked to functional cellular groups, including the pericarp, endosperm, embryo, and crease tissues, and identified novel marker genes linked to the clusters found. Our data also highlighted the utility of this technology to investigate subgenome biased spatial expression, highlighting expression differences between homeologs of key genes.

### 3.1 Gene Expression Clusters of the Endosperm Highlight Distinct Expression Zones

The physiological stages of wheat seed development have been established to take place in layers, where protein and starch accumulation differ between the outer and inner regions of the endosperm (Shewry et al. 2020), and transport of nutrients from the crease follows a gradient outwards towards the sub‐aleurone (Ugalde and Jenner 1990). Between approximately 8–21 DPA, the endosperm transitions from a liquid to a more solid state as this process of deposition takes place. The peak of grain filling is estimated to occur at 14–20 DPA, making this period significant to the yield and vigour of the resulting seed (Shewry et al. 2012). Li et al.'s (2025) analysis of the early developing wheat seed identified 10 gene expression clusters, including the inner and outer pericarp, the testa, the embryo and embryo surrounding regions, the aleurone and sub‐aleurone layers, the central cells of the starchy endosperm, the transfer cells surrounding endosperm, and the cavity fluid. From 4 to 12 DPA, the pericarp layers made up the largest area of the seed sections, and the endosperm was not yet cellularised, though multiple endosperm‐specific clusters could still be identified. In this study, the main viewpoint of spatial gene expression was of a longitudinal cross section parallel to the midline of the seed structure, which revealed a ring‐like shape of the aleurone and sub‐aleurone clusters. However, the other endosperm clusters did not appear to follow a concentric pattern from this view.

In the present study of the 14 DPA wheat seed, the endosperm region accounted for the largest area, highlighting the significance of the 12–14 DPA period for endosperm development and grain filling. Eight gene expression clusters were identified that largely corresponded with the clusters identified by Li et al. (2025), including the pericarp, sub‐aleurone, central endosperm, transfer cells and the embryo clusters. However, in the more mature grain only one pericarp cluster was identified, and the further development of the endosperm allowed for four distinct gene expression clusters to be delineated. These included the sub‐aleurone, the central endosperm, and the transfer cell clusters, as well as the inner endosperm cluster, which provides additional detail about gene expression in the developing endosperm, that hasn't yet been reported. It should also be noted that the ‘embryo surrounding region’ cluster was discounted from our data as this area was affected by tissue folding. Our study highlighted the opposing view of spatial gene expression, with longitudinal cross‐sections parallel to the dorsal and ventral sides of the seed structure. This revealed that the endosperm specific clusters formed concentric layers, following the oval shape of the seed and radiating inwards to encircle the crease. These gene expression patterns appear to align with the physiological development patterns of the seed, where different stages of protein, starch and nutrient deposition are taking place in layers across the endosperm at one time point. This study is the first time that four distinct regions of gene expression could be delineated from the endosperm during the peak of grain filling.

Interestingly, the gene expression matrix also revealed differences in overall expression between the different sides of the seed, both in the endosperm and pericarp tissues. We suggest that this could be a result of the position on the plant, with one side of the seed subject to more direct light resulting in higher biochemical activity and gene expression. This finding highlights the nuance of gene expression patterns that can be captured by spatial transcriptomics, as the gene expression of cells side by side can be compared and quantified in their entire spatial context.

### 3.2 Spatial Transcriptomics Reveals Subgenome Biased Expression Patterns of Key Genes

In polyploid plants, the contribution of subgenomes to different developmental and regulatory traits can be unbalanced, making subgenome specific gene expression an important aspect of transcriptomics analyses. Differences in expression between subgenome copies of various genes are reported in wheat. It is thought that unbalanced expression between homeologs is the first stage of neo‐functionalisation and enables functional specialisation and adaptability in polyploid plants (Ramirez‐Gonzalez et al. 2018). For instance, the B and D subgenomes are known to be more highly expressed in wheat overall (Ramirez‐Gonzalez et al. 2018), and subgenome D is reported to contribute disproportionately to abiotic and biotic stress responses (Zheng et al. 2022; Powell et al. 2016). Here, we report subgenome specific differences in expression between homologous gene triplicates of puroindoline‐b, metallothionein protein, and α‐amylase/subtilisin inhibitor. These findings were consistent with the literature as the subgenome B and D copies were more highly expressed than the subgenome A copies when they were present. However, there was no subgenome A copy for this metallothionein protein isoform encoded in the reference genome (Zhu et al. 2021), which could be an example of a gene lost due to unbalanced subgenome expression, making it vestigial.

Similarly, some subgenome specific spatial expression patterns have been reported in wheat (Wang et al. 2021) but are not yet well characterised as the capacity for detailed spatial transcriptomics has only recently advanced. Li et al. (2025) investigated subgenome biased spatial expression of the B3 transcription factor family in the 8 DPA seed section. They found higher expression of the B subgenome in the endosperm and aleurone, as well as greater expression of the subgenome B copy of *TaAB13‐B1* in the embryo at this developmental stage. Here, we reported subgenome specific spatial expression patterns for α‐amylase/subtilisin inhibitor, where the subgenome A copy was expressed at low levels in all tissues, compared to the subgenome B and D copies, which were significantly more highly expressed in the pericarp than any other tissue. Therefore, our study provides further evidence of differentiation between subgenomes at the spatial gene expression level. These findings are significant in the context of genetic research to optimise seed traits, as they highlight the utility of spatial transcriptomics for target gene analysis with both subgenome and tissue specificity.

### 3.3 Marker Genes Highlight Functions of Key Cellular Groups

The functions of particular cellular groups or tissue types, and how these components interact to influence important developmental traits, can be indicated by the genes most highly expressed in those tissues. In our study, strong marker genes were identified for the pericarp, crease, embryo, and transfer cell clusters, meaning they were highly expressed in those tissues and comparatively little in others. Additionally, marker genes were identified for the endosperm tissue, but were not specific to particular clusters. These findings have provided new information about the activity in these cellular groups during mid‐seed development.

The pericarp region is a structurally significant component of the seed and serves as an important protective layer for the endosperm and embryo tissues during development (Bechtel et al. 2009). The strongest marker genes identified in the pericarp were TraesCS7A03G1333700, encoding endochitinase, and TraesCS5A03G1295700, encoding a non‐specific lipid‐transfer protein. Endochitinases are known to play an important role in plant defence against fungal pathogens (Vaghela et al. 2022), while non‐specific lipid transfer proteins have roles in cell defence, cutin development, and structural development of cell membranes and cell walls (Salminen et al. 2016). Both marker genes are highly relevant to the role of the pericarp as the main structural and protective layer of the seed.

The crease is a region of the wheat seed that carries a high bacterial load (Robinson et al. 2016), pathogen defence and immune response genes are important to this tissue area. The strongest marker gene identified for the crease clusters in our gene expression matrix was TraesCS5A03G0651300 encoding induced stolen tip protein (TUB8). Induced stolen tip proteins are not yet characterised in wheat but have been reported in other plant species. A recent study in tomato identified an induced stolen tip protein as being a potential effect‐triggered immunity protein (Yu et al. 2021), and its expression in the crease could indicate that this gene could be involved in immune response and defence against pathogens in the wheat seed.

The embryo is a unique tissue in the seed with a distinct gene expression profile. The strongest marker gene identified for the embryo was TraesCS5B03G1068800, encoding cupincin protein. Cupincin also has not been studied in wheat; however, in rice, cupincin has been reported as a member of the cupin protein family and as having metallo‐protease activity, influencing seed vigour (Sreedhar and Kaul Tiku 2016). Therefore, this gene could also represent a potential target for optimising wheat seed performance.

The transfer cells cluster consists of cells encircling the crease, whose role is the transfer of proteins, starch, lipids and nutrients from the crease to the rest of the seed during development (Lopato et al. 2014). The strongest marker genes for this cluster were non‐specific lipid transfer protein genes and their high expression in this tissue during the peak of grain filling aligns with the anticipated expression patterns (Lopato et al. 2014). As these lipid transfer protein genes were some of the most highly expressed genes in the data overall, it is evident that they play a significant role in mid‐seed development and could be beneficial target genes for enhancing wheat yield. This represents an important avenue for further research.

The genes for α‐amylase/trypsin inhibitor proteins were identified as marker genes for the inner endosperm cluster, and were also expressed throughout the endosperm. These proteins are involved with pathogen resistance, inhibiting the hydrolytic enzymes of pests and pathogens, and they are reported to increase 3–10 fold during grain development (Geisslitz et al. 2021), their high expression at 14 DPA is consistent with the literature.

Lastly, starchy endosperm cells of the sub‐aleurone, central endosperm, and inner endosperm clusters make up the main part of the seed that is consumed by people; therefore, genes highly expressed in these tissues could be relevant to the nutritional and baking quality of wheat. The strongest marker genes identified for both the sub‐aleurone and central endosperm clusters were genes for α, β and γ‐gliadins, storage proteins common to the endosperm and that play a key role in gluten network formation (Chaudhary et al. 2022). These proteins are an important source of nitrogen and amino acids and, along with glutenins, impact the texture and quality of bread dough (Barak et al. 2014). Due to their contribution to the functional and nutritional properties of wheat, these genes are already well characterised in the literature; however, their importance as endosperm‐specific genes that are highly expressed during the peak of grain filling is further highlighted by our study.

Many of the marker genes identified represent important targets to better understand the tissue‐specific processes and interactions taking place during mid‐seed development. As different tissues and cellular groups contribute to different developmental traits, detail about the most highly expressed genes in those regions will allow for more targeted genetic research on those traits.

## 4 Conclusion

This study confirmed the spatial expression patterns of known tissue‐specific genes and identified novel marker genes for the pericarp, crease, embryo, and endosperm tissues of the developing wheat seed. These findings also highlighted the utility of spatial transcriptomics in studying subgenome differences, as we were able to distinguish subgenome‐biased expression and subgenome‐specific spatial expression patterns in a polyploid plant. Furthermore, this dataset represents a significant resource of spatial gene expression at 14 DPA and will support future research on the biological processes contributing to grain filling. As different tissues and cellular groups contribute differently to the functional and nutritional properties of seeds, spatial detail of gene expression during development provides valuable information for fine‐tuning targeted genetic research. The nutritional importance of seeds, particularly cereal grains, means that spatial gene expression data marks a significant advancement in our capacity to optimise food resources.

## 5 Material and Methods

### 5.1 Plant Growth Conditions and Sample Collection

Bobwhite SH98 26 seeds were germinated and grown in pots, 5 seeds per pot, in a glasshouse during winter in Brisbane, Australia. They were grown in media containing 2 g/L of Osmocote 3–4 month fertiliser and watered twice daily for 2 min at a time. Plants were monitored for the emergence of the first spikelet at 2.5 months of growth. Once the beginning of spikelet emergence could be observed, plants were tagged and dated, allowing for seed collection at 14 DPA, the stage of development where grain filling is at its peak (Kim et al. 2024).

Seeds were collected from the central third of the wheat ears, the glumes and lemmas were removed, and the seeds were immediately transferred into ambient temperature (liquid) Optimum Temperature Compound (OCT), in 1 × 1 cm containers. All seeds were placed on the bottom of the containers with the crease facing downwards, so they remained on the same plane. The containers were then transferred straight to dry ice where the OCT quickly froze. Sterile forceps were used to arrange the seeds and prevent them from floating up in the OCT before the block fully set. The time from removal of seeds from the plant to freezing was under 20 s.

### 5.2 Sample Preparation and Tissue Mounting

Seed sections were cut using a cryostat (CryoStar Nx70, Epredia). The cryostat chamber was cleaned with 70% ethanol and cooled to −22°C, and the blade to −18°C. The samples were placed in the chamber and allowed to equilibrate to −22°C for 30 min, as well as forceps and paint brushes for handling the tissue.

Stereo‐seq Capture Chips were removed from vacuum sealed bags and allowed to equilibrate to room temperature. To prepare the chips for mounting, they were rinsed with 100 μL of nuclease‐free water and then dried by power duster. Then 50 μL of 0.01% poly‐l‐lysine was added dropwise to the chip surface and left to stand for 10 min. The poly‐l‐lysine was then removed by power duster and the chips again rinsed with 100 μL of nuclease‐free water and dried. Chips were then placed in the cryostat chamber and cooled to −22°C.

Once cooled for 30 min, the samples were mounted onto the specimen disk holder and trimmed to the section to be analysed. Here, that was approximately one third of the way through the seeds when cutting longitudinally, where both the crease and embryo were included in the section. Tissue sections were cut at 20 μm thick and transferred to Stereo‐seq Capture chips using chilled forceps and brushes.

To mount the tissue sections, the chips were picked up within the cryostat chamber and warmed by pressing a finger to the back of the slide. The OCT thawed and the tissue section adhered to the chip surface. The slide was then dried at 37°C for 1 min and fixed in methanol at −20°C for 30 min.

### 5.3 Permeabilization Experiment

The permeabilization experiment was conducted first to determine the ideal permeabilization time for this tissue type, which would later be used for the transcriptomics experiment. This was done using the Stereo‐seq Permeabilization Set User Manual and following the manufacturer's guidelines with little modification (BGI, 211SC114) (Chen et al. 2022; Xia et al. 2022). In brief, four seed samples were each mounted onto a Stereo‐seq P chip and fixed (as described previously). After fixation, the samples were incubated at 37°C in 0.1% PR enzyme in 0.01 M HCl buffer, for 6, 12, 18, and 24 min respectively. The samples were then washed with 0.1× saline sodium citrate (SSC) solution. Reverse transcription was performed for 1 h at 42°C using the RT QC Mix, and then the tissue was removed from the chip surface by Tissue Removal Buffer.

Fluorescent images were taken of the chips at 5× and 10× objective lenses in the red channel. Based on the images taken (Figure S22), 12 min produced the brightest image with the least degradation and was determined to be the optimal permeabilization time for wheat grain tissue.

### 5.4 Transcriptomics Experiment

The transcriptomics experiment was conducted using the Stereo‐seq Transcriptomics Set User Manual and following the manufacturer's instructions with little modification (BGI, 211SC114) (Chen et al. 2022; Xia et al. 2022). In brief, four seed samples were each mounted onto a Stereo‐seq T chip and fixed (as described previously). After tissue fixation, the sections were stained for fluorescent imaging by the Qubit ssDNA reagent. Fluorescent images were taken of each chip using the 10× objective lens in both the FITC channel and DAPI channel.

The samples were then permeabilised in 0.1% PR enzyme in 0.01 M HCl buffer for 12 min at 37°C, so that the RNA could be released from the tissue sections and captured on the chips' surface. Reverse transcription was then performed overnight at 37°C. The next day, Tissue Removal Buffer was used to remove the tissue from the chips' surface and the remaining cDNA was released by cDNA Release Buffer for 3 h.

The collected cDNA was then used for library construction by the Stereo‐seq Library Preparation kit. The resulting libraries were pooled and sequenced by whole genome sequencing using the T7 PE100 flow cell.

During the protocol, challenges arose with the tissue mounting step, resulting in some folding and breaking of the tissue sections. We proceeded with the most intact tissue sections for the subsequent analyses.

### 5.5 SAW Pipeline

The SAW software suite (https://github.com/BGIResearch/SAW), with default parameters, was used to map the raw sequencing reads to the wheat reference genome IWGSC CS RefSeq v2.1 (Zhu et al. 2021). Reads that could be uniquely mapped to coding regions of the genome were counted, and the coordinate identity (CID) of each read was used to place it spatially in its original position on the chips. This data was overlayed with the fluorescent microscope images of the tissue sections before permeabilization, so that reads could be mapped to their spatial location within the tissue. The SAW software suite was then used to generate a matrix of expression count of all genes expressed at each spot on the chips. To handle the sparseness of the gene expression within each spot (i.e., only a subset of genes are captured per spot) adjacent spots could be grouped into different bin levels, such as Bin200 for 200 × 200 spots, for analysis at varying resolutions. It was determined that Bin50 was the ideal resolution for this data.

Once the gene expression matrices were generated, one tissue section was deemed to be the highest quality replicate compared to the others which exhibited damage due to challenges during the tissue mounting step. The chip containing the most intact replicate was designated as the focus for the subsequent analysis, while the remaining chips were referenced to provide replication where possible.

### 5.6 Clustering and Marker Gene Discovery

The Stereopy library was used to generate gene clusters and marker genes at the desired Bin level, Bin50. The gene expression matrices were first filtered to remove bins with low MID counts. Due to the expression levels of genes within the tissue boundaries and expression levels of the background noise outside the tissue boundaries, a threshold of a minimum 30 MID counts per bin was selected for the focus chip, and a threshold of between a minimum 30 and 50 MID counts was selected for the replicate chips. Filtering the data by expression levels also increased the computational efficiency of the analysis and allowed for downstream generation of spatial neighbours clusters.

The expression data was normalised by the log1p method (Booeshaghi and Pachter 2021) and then underwent principal component analysis (PCA). A neighbourhood graph of bins was then generated using the PCA representation of the expression matrix, followed by a spatial neighbours graph. These graphs were then embedded into two dimensions for ease of visualisation using UMAP.

Leiden clustering was then undertaken using the spatial neighbours data. This network analysis tool grouped bins based on the expression profile of the genes at each location. All transcripts captured on the chip were included in the network, resulting in 11–12 distinct gene expression clusters being identified for each chip. Overlaying the spatial Leiden clusters with the fluorescent microscope images of the tissue allowed us to link eight clusters to functional cellular groups and determine that some clusters could be discarded as background noise or as a result of tissue folding issues. This was due to their location outside the tissue boundary or a pattern following the shape of the folded areas that did not align with any particular cell or tissue type.

Lastly, marker gene candidates were identified for each gene expression cluster, using a *t*‐test on the raw MID count data, to compute a ranking of differentially expressed genes across clusters. This analysis was repeated for all chips analysed, and marker genes identified for their tissue type on multiple chips were recorded.

### 5.7 Investigation of Known Tissue‐Specific Genes

Known tissue specific genes were identified from the literature, including genes expected or known to be specifically expressed in the endosperm, pericarp, and embryo tissues, and Blastn analysis was used to identify subgenome homeologs (Sayers et al. 2024). Their spatial expression patterns and subgenome specific expression were investigated using our gene expression matrix and the Stereomap app. The expression of homologous gene triplicates was visualised in groups and individually, in order to capture their overall expression and compare between subgenomes.

Furthermore, a sub‐sample of 5% of bins from each cluster was randomly selected and used to undertake differential expression analyses. The bins were selected from clusters generated using the log1p normalised expression data, and those bin IDs were then used to extract the unnormalized MID count data from those locations in the chip. The raw count data for the tissue specific target genes at each randomly sampled bin ID (location) were then used for differential expression analysis to quantify the differences in expression of homologous gene copies across different clusters (tissue types) and also to quantify the differences in overall expression between pairs of genes within homologous gene triplicates. Lastly, we also compared expression of individual α‐amylase/subtilisin inhibitor homeologs across clusters, as these genes appeared to exhibit spatial subgenome biased expression, based on the Stereomap images (Figure 4D–F).

To determine the statistical significance of the differences in gene expression, we used an approximate (Monte Carlo) permutation test. This test was chosen as the highly zero‐inflated spatial expression data would violate the normality assumption of a standard *t*‐test and produce poor results under an equivalent rank‐based test. In the approximate permutation test, 500 000 random permuted samples of the two sets of gene expression data being tested were generated, which provided an estimate of the *p*‐value. Binomial theory was then applied to give a 99.9% confidence interval for the true *p*‐value. The estimated *p*‐values were then adjusted by a Bonferroni multiple testing correction factor of 264, the total number of tests conducted, resulting in values that we have a high level of confidence the true adjusted *p*‐values were less than, as reported in Tables S1–S9.

## Author Contributions

R.J.H. conceived and supervised the project. T.M., R.S., X.S., and K.L.L. conducted the experiments. T.M., D.K., L.M., and A.M. conducted the analysis. T.M. wrote the manuscript with input from D.K., R.S., and R.J.H. All authors read the final version of the paper.

## Conflicts of Interest

The authors declare no conflicts of interest.

## Supporting information


**Data S1:** pbi70351‐sup‐0001‐DataS1.zip.

## Data Availability

The high‐throughput data that supports the findings of this study are openly available in Gene Expression Omnibus at https://www.ncbi.nlm.nih.gov/geo/query/acc.cgi?acc=GSE298021, accession number GSE298021. The scripts used have been uploaded to GitHub (https://github.com/tori‐millsteed/Wheat_seed_spatial_transcriptomics_Millsteed2025).
