# Supplementary material for: Spatial Transcriptomics of Developing Wheat Seed Reveals Concentric Gene Expression Zones and Subgenome Biased Expression of Key Genes
Source: Plant Biotechnol J. 2025 Sep 4;23(12):5934–49. doi: 10.1111/pbi.70351 (PMC12665067; doi:10.1111/pbi.70351)
Supplement: Supplementary file 1 — Data S1: pbi70351‐sup‐0001‐DataS1.zip. [file PBI-23-5934-s001.zip › Captions for Tables S1-S9.docx]

Captions for Tables S1-S9

**Table S1.** Approximate permutation tests comparing pairs of genes within homologous gene triplicates, to determine significant differences in expression. This table represents data analysed from chip D02266B1 (replicate 1), and includes the common names of genes being compared, the observed difference in means, the estimated p-value, the adjusted p-value (by a Bonferroni multiple testing correction factor of 264), the number of replicates used by the permutation test, and the gene names from the reference genome, ie the gene IDs.

**Table S2.** Approximate permutation tests comparing the expression of grouped homologous gene triplicates across different tissue types (spatial gene expression clusters) to determine significant differences in spatial expression. This table represents data analysed from chip D02266B1 (replicate 1), and includes the common names of the gene triplicates to be analysed, the names of the cluster groups to be compared, the observed difference in means, the estimated p-value, the adjusted p-value (by a Bonferroni multiple testing correction factor of 264), the number of replicates used by the permutation test, the gene names from the reference genome, ie the gene IDs, and the cluster IDs.

**Table S3.** Approximate permutation tests comparing pairs of homologous α-amylase/subtilisin inhibitor genes, across different tissue types (spatial gene expression clusters), to determine significant differences in spatial expression. This table represents data analysed from chip D02266B1 (replicate 1), and includes the common names of the genes being compared, the names of the cluster groups to be compared, the observed difference in means, the estimated p-value, the adjusted p-value (by a Bonferroni multiple testing correction factor of 264), the number of replicates used by the permutation test, the gene names from the reference genome, ie the gene IDs, and the cluster IDs.

**Table S4.** Approximate permutation tests comparing pairs of genes within homologous gene triplicates, to determine significant differences in expression. This table represents data analysed from chip D02266A4 (replicate 2), and includes the common names of genes being compared, the observed difference in means, the estimated p-value, the adjusted p-value (by a Bonferroni multiple testing correction factor of 264), the number of replicates used by the permutation test, and the gene names from the reference genome, ie the gene IDs.

**Table S5.** Approximate permutation tests comparing the expression of grouped homologous gene triplicates across different tissue types (spatial gene expression clusters) to determine significant differences in spatial expression. This table represents data analysed from chip D02266A4 (replicate 2), and includes the common names of the gene triplicates to be analysed, the names of the cluster groups to be compared, the observed difference in means, the estimated p-value, the adjusted p-value (by a Bonferroni multiple testing correction factor of 264), the number of replicates used by the permutation test, the gene names from the reference genome, ie the gene IDs, and the cluster IDs.

**Table S6.** Approximate permutation tests comparing pairs of homologous α-amylase/subtilisin inhibitor genes, across different tissue types (spatial gene expression clusters), to determine significant differences in spatial expression. This table represents data analysed from chip D02266A4 (replicate 2), and includes the common names of the genes being compared, the names of the cluster groups to be compared, the observed difference in means, the estimated p-value, the adjusted p-value (by a Bonferroni multiple testing correction factor of 264), the number of replicates used by the permutation test, the gene names from the reference genome, ie the gene IDs, and the cluster IDs.

**Table S7.** Approximate permutation tests comparing pairs of genes within homologous gene triplicates, to determine significant differences in expression. This table represents data analysed from chip D02263D4 (replicate 3), and includes the common names of genes being compared, the observed difference in means, the estimated p-value, the adjusted p-value (by a Bonferroni multiple testing correction factor of 264), the number of replicates used by the permutation test, and the gene names from the reference genome, ie the gene IDs.

**Table S8.** Approximate permutation tests comparing the expression of grouped homologous gene triplicates across different tissue types (spatial gene expression clusters) to determine significant differences in spatial expression. This table represents data analysed from chip D02263D4 (replicate 3), and includes the common names of the gene triplicates to be analysed, the names of the cluster groups to be compared, the observed difference in means, the estimated p-value, the adjusted p-value (by a Bonferroni multiple testing correction factor of 264), the number of replicates used by the permutation test, the gene names from the reference genome, ie the gene IDs, and the cluster IDs.

**Table S9.** Approximate permutation tests comparing pairs of homologous α-amylase/subtilisin inhibitor genes, across different tissue types (spatial gene expression clusters), to determine significant differences in spatial expression. This table represents data analysed from chip D02263D4 (replicate 3), and includes the common names of the genes being compared, the names of the cluster groups to be compared, the observed difference in means, the estimated p-value, the adjusted p-value (by a Bonferroni multiple testing correction factor of 264), the number of replicates used by the permutation test, the gene names from the reference genome, ie the gene IDs, and the cluster IDs.
