# Supplementary material for: Spatial Transcriptomics of Developing Wheat Seed Reveals Concentric Gene Expression Zones and Subgenome Biased Expression of Key Genes
Source: Plant Biotechnol J. 2025 Sep 4;23(12):5934–49. doi: 10.1111/pbi.70351 (PMC12665067; doi:10.1111/pbi.70351)
Supplement: Supplementary file 1 — Data S1: pbi70351‐sup‐0001‐DataS1.zip. [file PBI-23-5934-s001.zip › pbi70351-sup-0001-DataS1.pdf]

Supporting Data

Key Metrices

Total Reads	780,145,171	100%
Valid CID Reads	570,305,187	73.1%
Clean Reads	542,585,536	95.1%
Uniquely Mapped Reads	36,415,138	6.7%
Transcriptome	16,611,992	45.6%
Unique Reads	5,741,511	34.6%
Sequencing Saturation	10,870,481	65.4%
Unannotated Reads	19,803,146	54.4%
Multi-Mapped Reads	493,843,278	91.0%
Unmapped Reads	12,327,120	2.3%
Non-Relevant Short Reads	27,719,651	4.9%
Invalid CID Reads	204,300,199	26.2%
Discarded MID Reads	5,539,785	0.7%

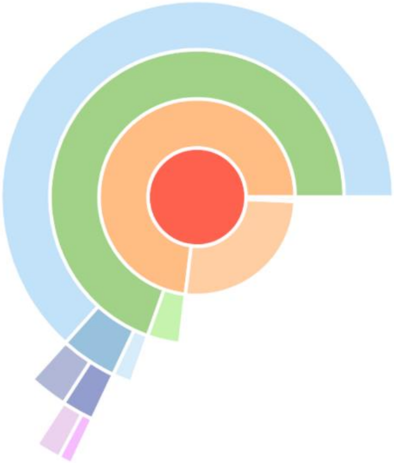

Figure S1. SAW analytical report key metrices for chip 1 (chip ID D02266B1)

Tissue

DNB Under Tissue	92,046,597
mRNA-Captured DNBs Under Tissue	3,273,120
Genes Under Tissue	60,877
Number of MID Under Tissue Coverage	4,244,343
Fraction MID in Spots Under Tissue	73.92%
Reads Under Tissue	365,103,798
Fraction Reads in Spots Under Tissue	63.40%

Figure S2. SAW analytical report tissue data for chip 1 (chip ID D02266B1)

Key Metrices

Total Reads	1,110,260,180	100%
Valid CID Reads	857,718,889	77.3%
Clean Reads	824,477,468	96.1%
Uniquely Mapped Reads	79,536,921	9.6%
Transcriptome	52,673,637	66.2%
Unique Reads	14,320,878	27.2%
Sequencing Saturation	38,352,759	72.8%
Unannotated Reads	26,863,284	33.8%
Multi-Mapped Reads	734,027,333	89.0%
Unmapped Reads	10,913,214	1.3%
Non-Relevant Short Reads	33,241,421	3.9%
Invalid CID Reads	244,232,972	22.0%
Discarded MID Reads	8,308,319	0.7%

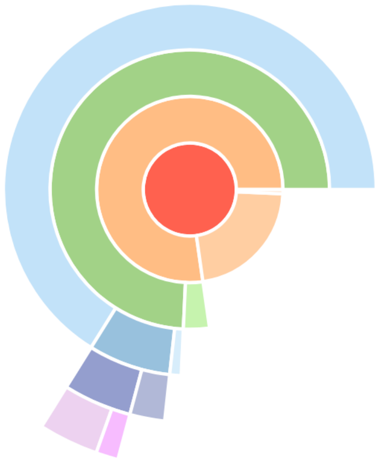

Figure S3. SAW analytical report key metrices for chip 2 (chip ID D02266A4)

Tissue

DNB Under Tissue	90,326,917
mRNA-Captured DNBs Under Tissue	7,382,161
Genes Under Tissue	60,916
Number of MID Under Tissue Coverage	11,599,308
Fraction MID in Spots Under Tissue	81.00%
Reads Under Tissue	507,757,235
Fraction Reads in Spots Under Tissue	58.63%

Figure S4. SAW analytical report tissue data for chip 2 (chip ID D02266A4)

Key Metrices

Total Reads	1,324,226,135	100%
Valid CID Reads	1,010,302,264	76.3%
Clean Reads	973,284,122	96.3%
Uniquely Mapped Reads	49,272,216	5.1%
Transcriptome	22,842,830	46.4%
Unique Reads	6,036,443	26.4%
Sequencing Saturation	16,806,387	73.6%
Unannotated Reads	26,429,386	53.6%
Multi-Mapped Reads	911,428,823	93.6%
Unmapped Reads	12,583,083	1.3%
Non-Relevant Short Reads	37,018,142	3.7%
Invalid CID Reads	305,429,088	23.1%
Discarded MID Reads	8,494,783	0.6%

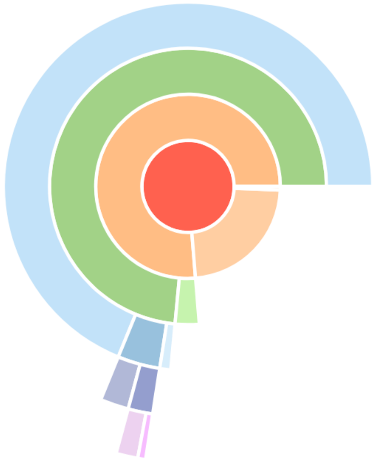

Figure S5. SAW analytical report key metrices for chip 3 (chip ID D02263D4)

Tissue

DNB Under Tissue	85,660,333
mRNA-Captured DNBs Under Tissue	3,625,784
Genes Under Tissue	59,190
Number of MID Under Tissue Coverage	5,218,664
Fraction MID in Spots Under Tissue	86.45%
Reads Under Tissue	574,757,616
Fraction Reads in Spots Under Tissue	56.42%

Figure S6. SAW analytical report tissue data for chip 3 (chip ID D02263D4)

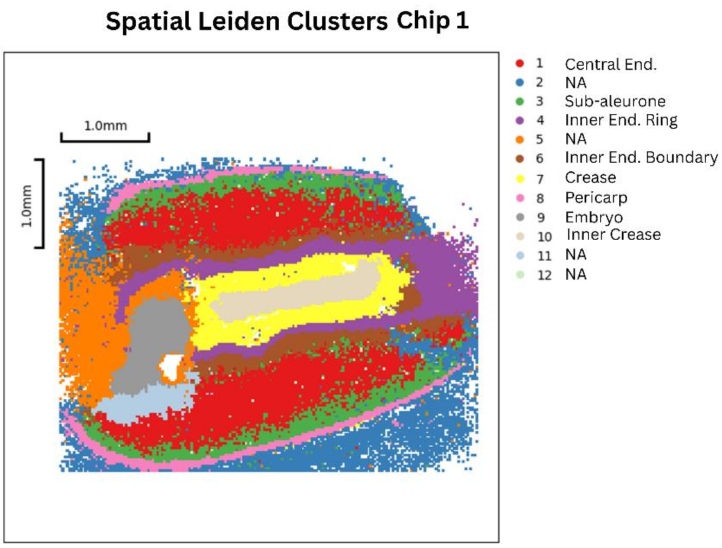

Figure S7: Spatial Leiden clusters for chip 1 (zoomed in to view only one seed section)

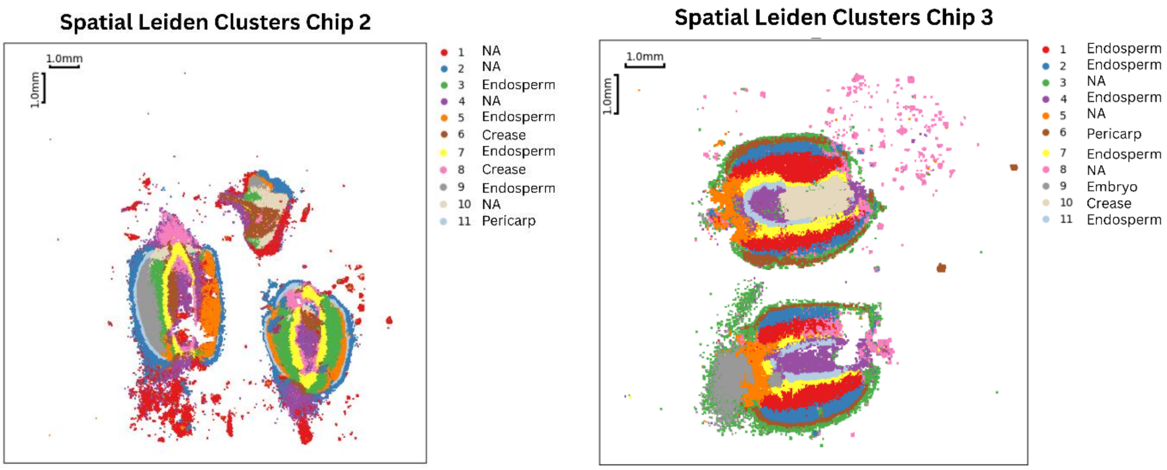

Figure S8: Spatial Leiden clusters of chips 2 and 3, no zoom applied

TaNAC019

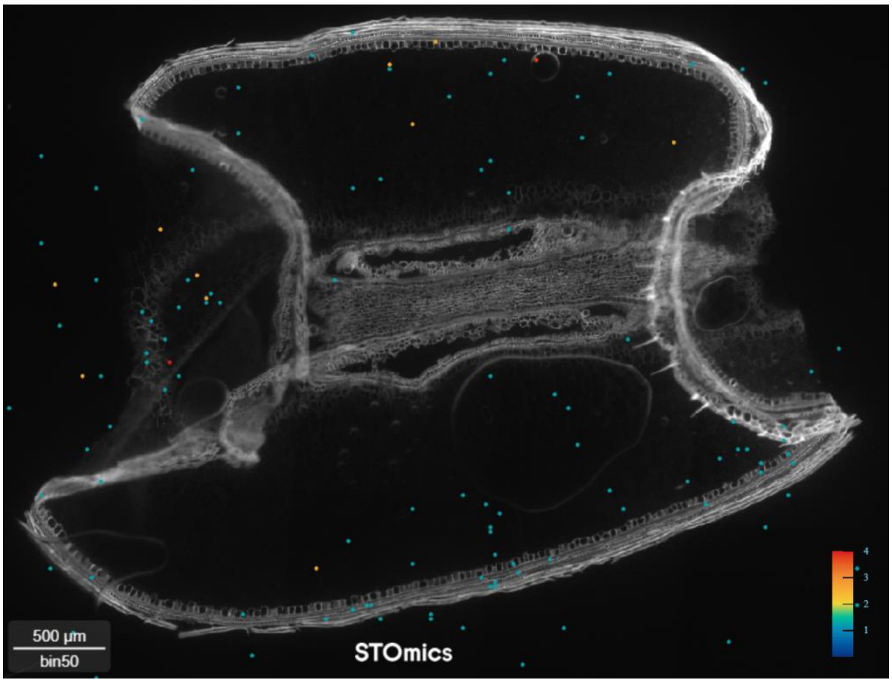

Figure S9. TaNAC019 spatial expression

TabZIP28

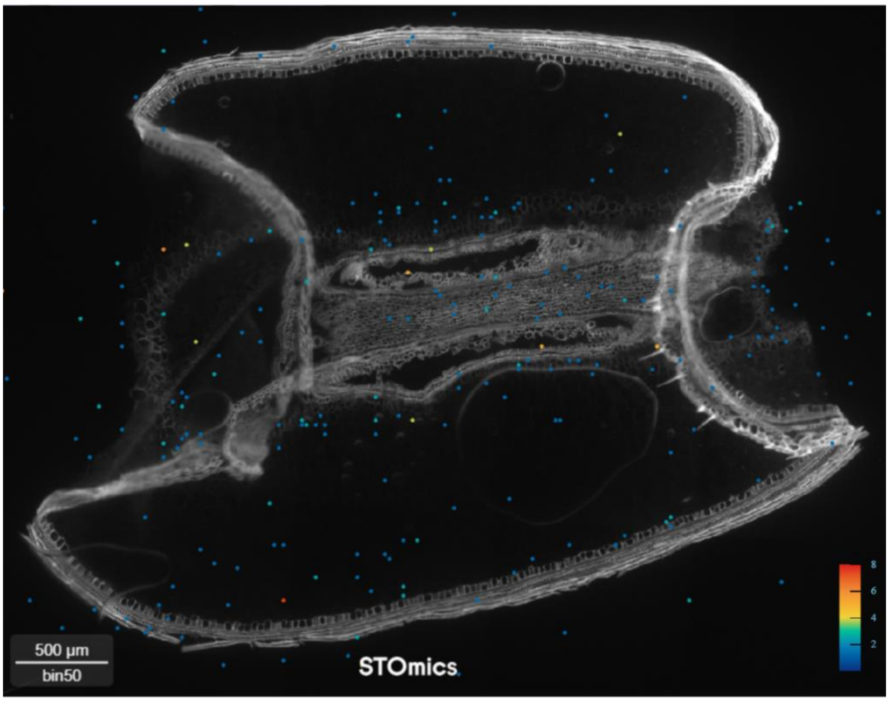

Figure S10. TabZIP28 spatial expression

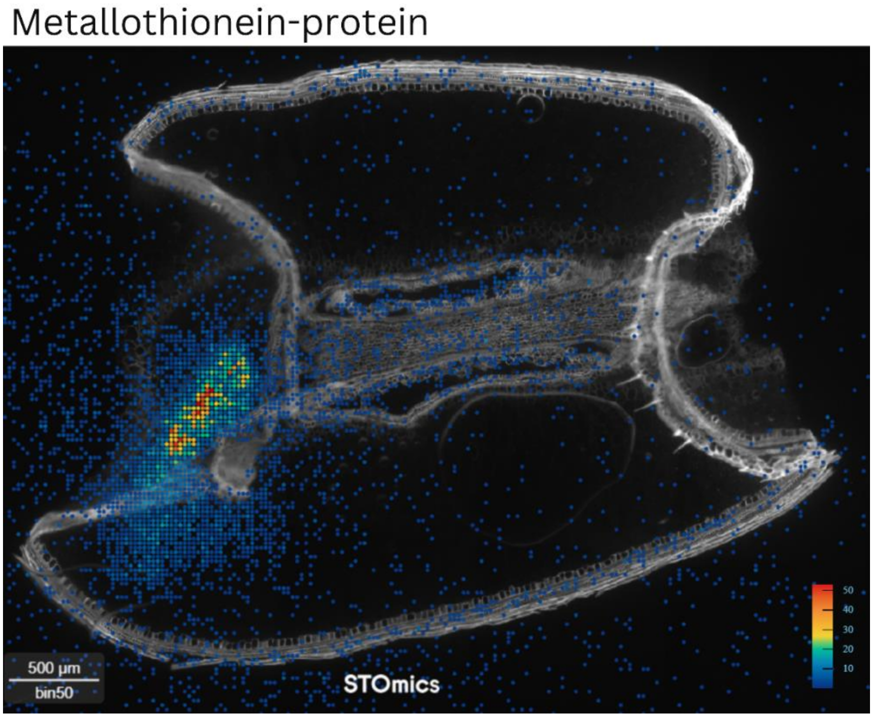

Figure S11. Metallothionein-protein spatial expression

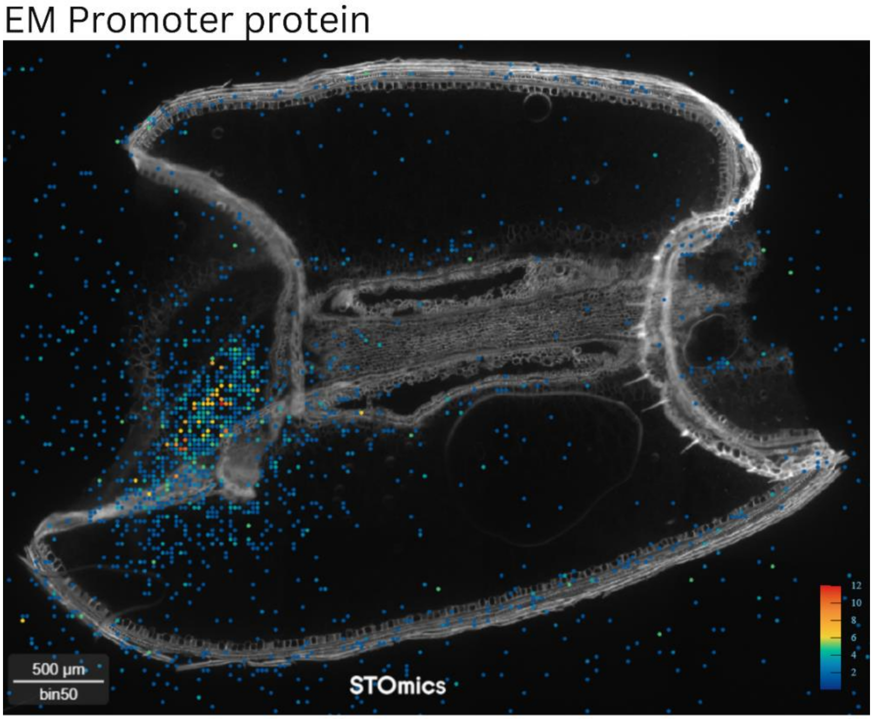

Figure S12. EM promoter protein spatial expression

Pyruvate orthophosphate dikinase

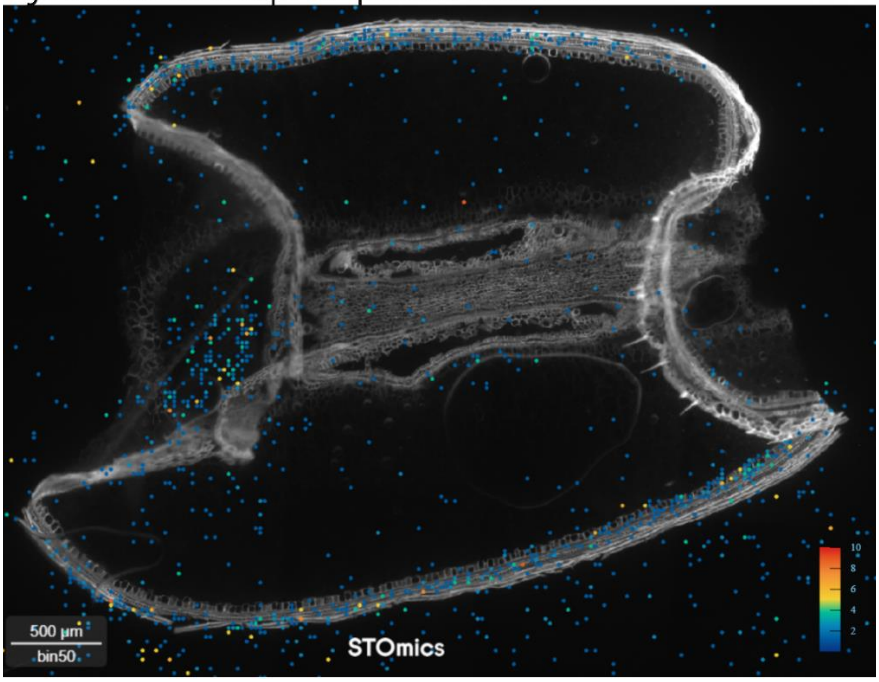

Figure S13. Pyruvate orthophosphate dikinase spatial expression

Subgenome A Subgenome B Subgenome D

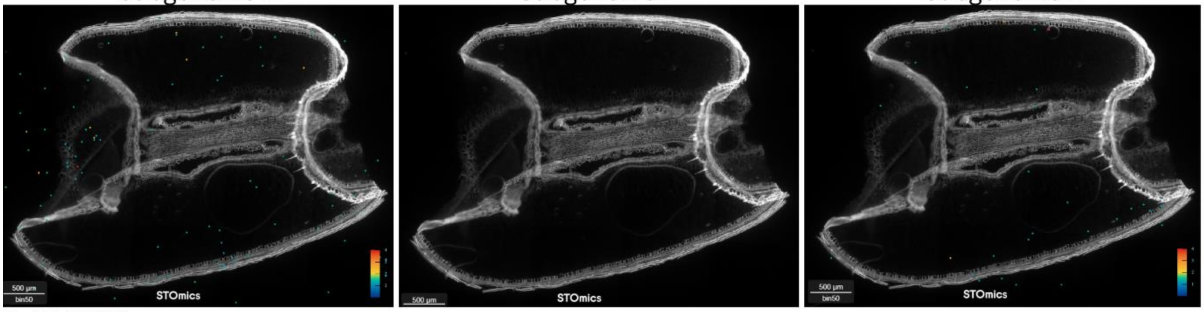

TaNAC019

Figure S14. TaNAC019 subgenome specific expression

Subgenome A Subgenome B Subgenome D

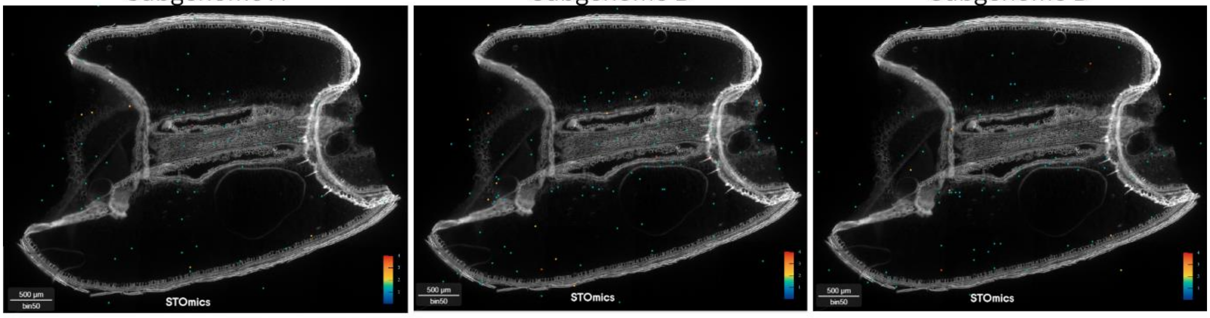

TabZIP28

Figure S15. TabZIP28 subgenome specific expression

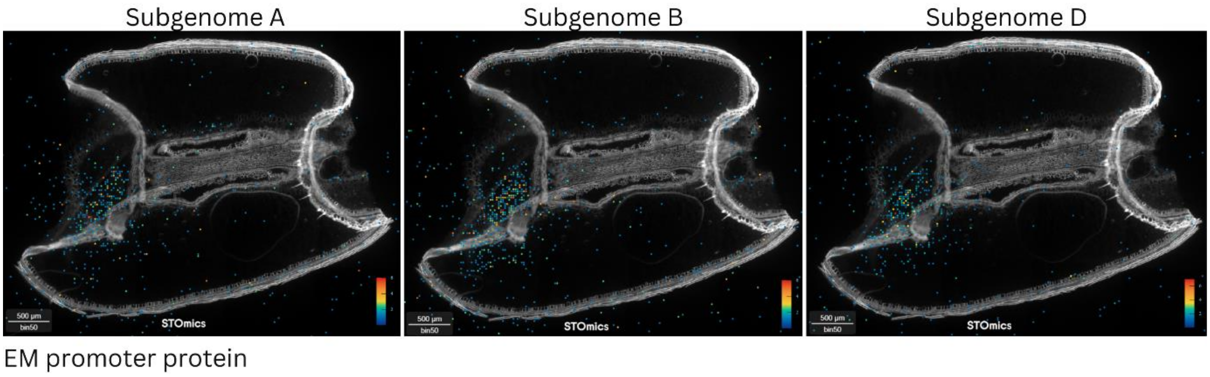

Figure S16. EM promoter protein subgenome specific expression

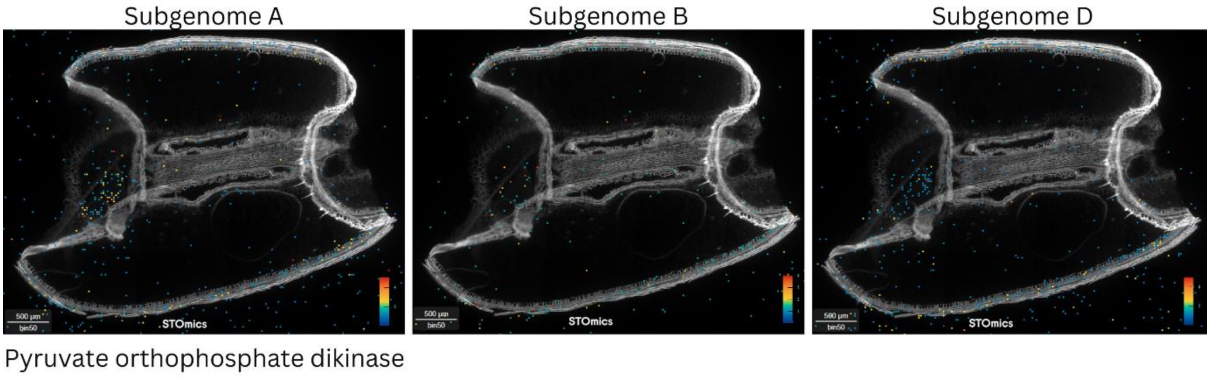

Figure S17. Pyruvate orthophosphate dikinase subgenome specific expression

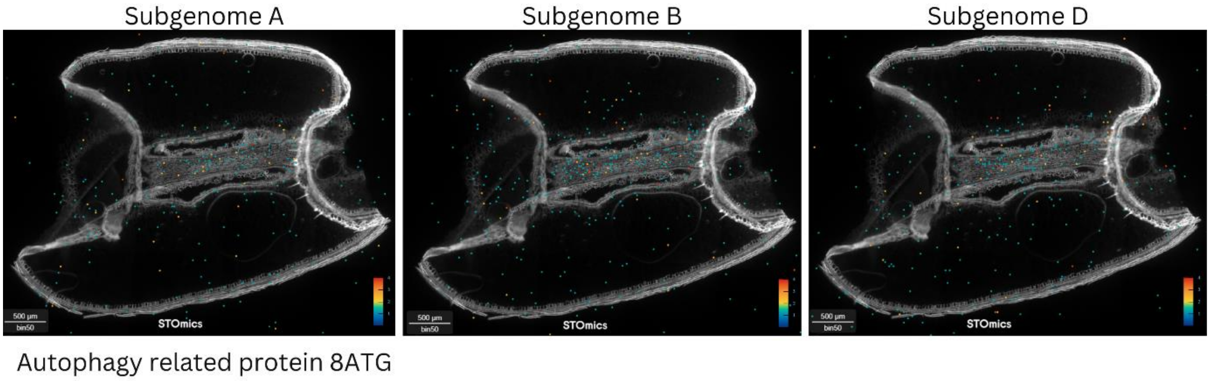

Figure S18. Autophagy related protein ATG8 subgenome specific expression

Table S1. Reported and observed spatial expression patterns of known tissue-specific genes in a 14 days post anthesis (DPA) wheat seed section. Table includes gene name and/or function, NCBI gene IDs, subgenome of homeologs, the reported spatial expression pattern from the literature , the observed spatial expression pattern in the data presented, and the overall homeolog expression (in MID counts) in the replicate seed sections analysed.

Gene name/ function	Gene ID	Sub-genome	Expected spatial expression	Observed spatial expression in our data	Overall homeolog expression (MID count)		
					Rep 1	Rep 2	Rep 3
Puroindoline-B	*LOC123101925*	A	Endosperm/ aleurone	Endosperm/ aleurone	2836	15,021	3817
	LOC543308	B			7387	35,537	10,749
	LOC100125699	D			24,634	97,257	34,577
TaNAC019	*LOC123057832*	A	Endosperm	Endosperm	238	373	226
	LOC123064838	B			0	0	0
	LOC123073994	D			82	262	86
TabZIP28	*LOC123187748*	A	Endosperm	Endosperm/ crease	153	219	146
	LOC123043995	B			287	383	200
	LOC123051864	D			205	242	148
Metallothionein-protein		A	Embryo	Embryo/ pericarp/ crease	0	0	0
	LOC123104584	B			2131	585	785
	LOC123179983	D			21,397	8362	9896
EM promoter protein	*LOC543476*	A	Embryo	Embryo/ pericarp/ crease	1543	1617	893
	LOC543084	B			2100	1292	819
	LOC123182837	D			1439	894	550
α-amylase/ subtilisin inhibitor	*LOC123185730*	A	Pericarp	Endosperm	123	458	109
	LOC123041664	B		Pericarp/ endosperm	4286	13,229	3443
	LOC123049628	D			2730	13,649	4056
Pyruvate orthophosphate dikinase	*LOC123055206*	A	Pericarp	Pericarp/ embryo/ endosperm	1380	3111	1038
	LOC123132411	B			609	1658	579
	LOC123181969	D			1418	2625	884
Autophagy related protein ATG8	*LOC123188447*	A	Pericarp	Crease/ endosperm/ pericarp	533	958	371
	LOC123044698	B			711	1021	432
	LOC542962	D			690	1123	515

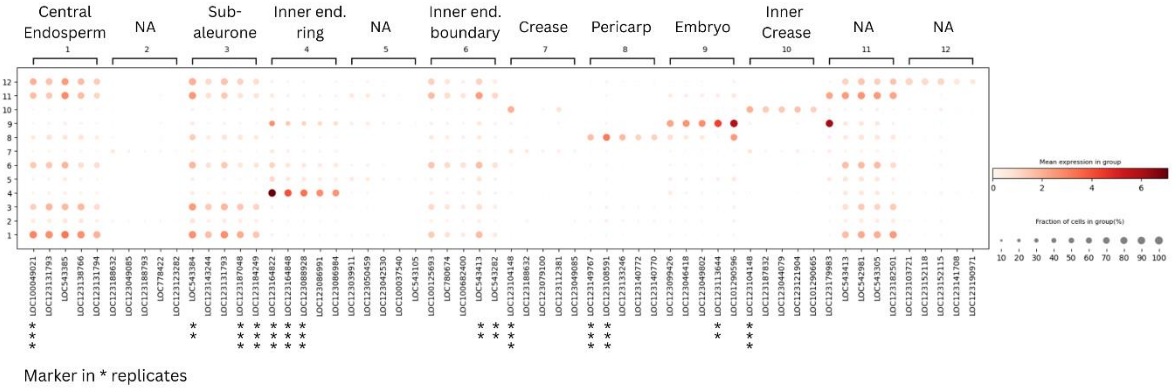

Figure S19. Marker genes plot of chip 1, with NCBI gene IDs

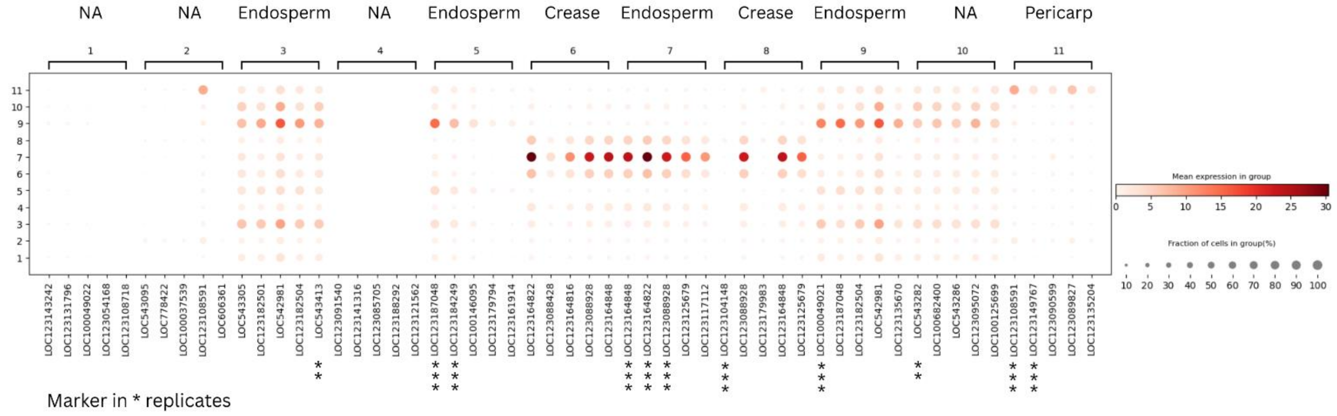

Figure S20: Marker genes plot of chip 2, with NCBI gene IDs

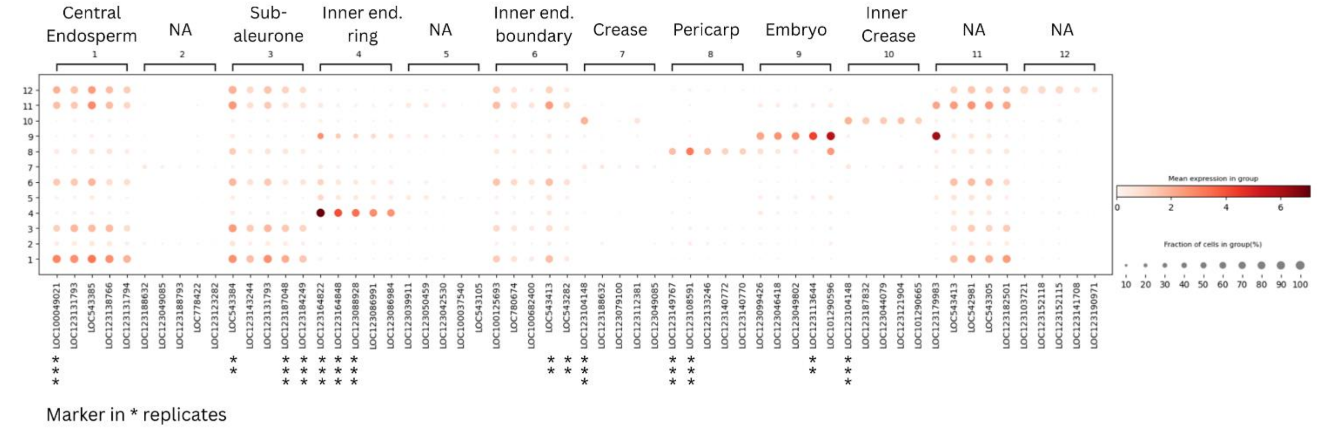

Figure S21: Marker genes plot of chip 3, with NCBI gene IDs

Table S2. Marker gene candidates identified for each gene expression cluster. Including the cluster name, the IWGSC Chinese Spring Refseq v2.1 gene ID, the gene name/function, and the number of times a gene was identified as a marker candidate for the respective cluster across the three replicate seed sections analysed (*).

Cluster	Gene ID	Gene Name/Function	Marker gene for this tissue area in * replicates
1) Central endosperm	LOC100049021	alpha/beta-gliadin	***
	LOC123131793	alpha/beta-gliadin	*
	LOC543385	alpha/beta-gliadin	*
	LOC123138766	alpha/beta-gliadin	*
	LOC123131794	alpha/beta-gliadin	*
3) Sub-aleurone	LOC543384	gamma-gliadin	**
	LOC123143244	alpha/beta-gliadin	*
	LOC123131793	alpha/beta-gliadin	*
	LOC123187048	gamma-gliadin	***
	LOC123184249	vacuolar protein sorting-associated protein 60.1	***
4) Transfer cells	LOC123164822	uncharacterised	***
	LOC123164848	uncharacterised	***

	LOC123088928	uncharacterised	***
	LOC123086991	uncharacterised	*
	LOC123086984	uncharacterised	*
6) Inner endosperm	LOC100125693	alpha-amylase inhibitor 0.19	*
	LOC780674	alpha-amylase inhibitor 0.19-like	*
	LOC100682400	alpha-amylase/trypsin inhibitor CM2-like	*
	LOC543413	purothionin A	**
	LOC543282	alpha-amylase/trypsin inhibitor CM1-like	**
7) Outer crease	LOC123104148	induced stolen tip protein TUB8	***
	LOC123188632	photosystem II protein D1-like	*
	LOC123079100	BURP domain-containing protein 3	*
	LOC123112381	skin secretory protein xP2	*
	LOC123049085	photosystem II protein D1-like	*
8) Pericarp	LOC123108591	non-specific lipid-transfer protein 2P-like	***
	LOC123149767	26 kDa endochitinase 2	***
	LOC123133246	glycine rich cell wall structural protein	*
	LOC123140772	glycine rich cell wall structural protein	*
	LOC123140770	glycine rich cell wall structural protein	*
9) Embryo	LOC123099426	oleosin 18 kDa-like	*
	LOC123046418	oleosin 16 kDa	*
	LOC123049802	oleosin 16 kDa-like	*
	LOC123113644	cupincin	**
	LOC101290596	63 kDa globulin-like protein	*
10) Inner crease	LOC123104148	induced stolen tip protein TUB8	***
	LOC123187832	uncharacterised	*
	LOC123044079	suppressor of disruption of TFIIS-like	*
	LOC123121904	predicted GPI-anchored protein 58	*
	LOC101290665	gibberellin 2-beta-dioxygenase 3	*

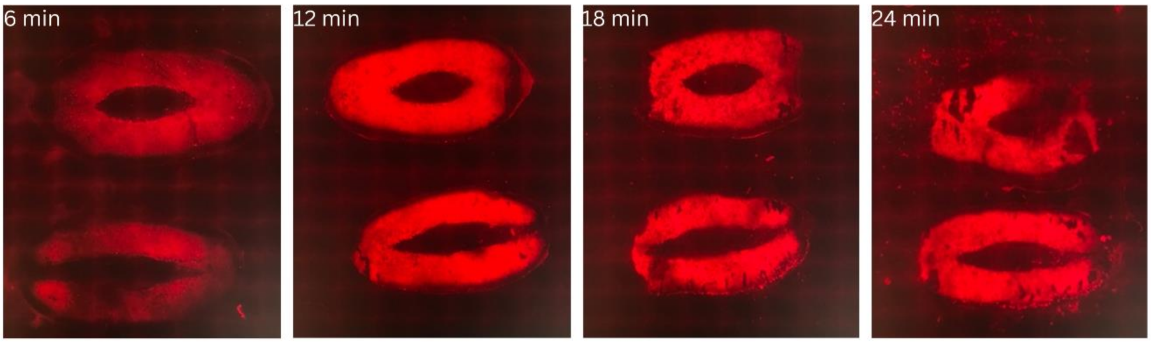

Figure S22. Permeabilization results after 6, 12, 18 and 24 min of treatment with permeabilization reagent
